# Fine-tuning of microRNA-mediated repression of mRNA by splicing-regulated and highly repressive microRNA recognition element

**DOI:** 10.1186/1471-2164-14-438

**Published:** 2013-07-03

**Authors:** Cheng-Tao Wu, Chien-Ying Chiou, Ho-Chen Chiu, Ueng-Cheng Yang

**Affiliations:** 1 Institute of Biomedical Informatics, National Yang-Ming University, No.155, Sec.2, Linong Street, Taipei 11221, Taiwan, ROC; 2 Biomedical Technology and Device Research Labs (BDL), Industrial Technology Research Institute (ITRI), No.195, Sec. 4, Chung Hsing Rd., Chutung, Hsinchu 31040, Taiwan, ROC; 3 Center for Systems and Synthetic Biology, National Yang-Ming University, No.155, Sec.2, Linong Street, Taipei 11221, Taiwan, ROC; 4 Bioinformatics Consortium of Taiwan core facility, Taipei, Taiwan, ROC

**Keywords:** microRNA, Alternative splicing, MicroRNA recognition element, Repressive ratio

## Abstract

**Background:**

MicroRNAs are very small non-coding RNAs that interact with microRNA recognition elements (MREs) on their target messenger RNAs. Varying the concentration of a given microRNA may influence the expression of many target proteins. Yet, the expression of a specific target protein can be fine-tuned by alternative cleavage and polyadenylation to the corresponding mRNA.

**Results:**

This study showed that alternative splicing of mRNA is a fine-tuning mechanism in the cellular regulatory network. The splicing-regulated MREs are often highly repressive MREs. This phenomenon was observed not only in the hsa-miR-148a-regulated DNMT3B gene, but also in many target genes regulated by hsa-miR-124, hsa-miR-1, and hsa-miR-181a. When a gene contains multiple MREs in transcripts, such as the VEGF gene, the splicing-regulated MREs are again the highly repressive MREs. Approximately one-third of the analysable human MREs in MiRTarBase and TarBase can potentially perform the splicing-regulated fine-tuning. Interestingly, the high (+30%) repression ratios observed in most of these splicing-regulated MREs indicate associations with functions. For example, the MRE-free transcripts of many oncogenes, such as N-RAS and others may escape microRNA-mediated suppression in cancer tissues.

**Conclusions:**

This fine-tuning mechanism revealed associations with highly repressive MRE. Since high-repression MREs are involved in many important biological phenomena, the described association implies that splicing-regulated MREs are functional. A possible application of this observed association is in distinguishing functionally relevant MREs from predicted MREs.

## Background

MicroRNAs, which are very short non-coding RNAs of 21–23 nucleotides in length, play an important role in the regulation of gene expression, disease progression [1], development [2,3], differentiation [4] and cancer [5]. This regulatory process is mainly mediated by targeting microRNA recognition elements (MREs) in the 3′ untranslated region (3′UTR) [6] or the coding region [7,8] of one or more messenger RNAs (mRNAs). As a result, either the targeted messenger RNAs are cleaved or the translation of transcripts is repressed [9].

Legendre *et al*. [10] hypothesized that some of the shorter MRE-free transcripts may escape from microRNA-mediated inhibition and further observed that alternative cleavage and polyadenylation (APA) reduces the amount of MRE-containing isoforms in the corresponding microRNA-expressed tissue. This finding is consistent with the observation that approximately two-thirds of the MRE-containing genes have isoforms in the 3′-UTRs [11]. This hypothesis is also supported by findings that activation of primary murine CD4+ T lymphocytes is associated with an increased number of isoforms that lack MREs [12]. Restated, these MRE-free isoforms have escaped microRNA-mediated inhibition. Furthermore, in cancer cells, oncogene activation is often accompanied by APA of mRNAs, which can cause widespread loss of 3′UTR repressive elements [13]. The above reports reveal not only that APA is a regulatory mechanism of microRNA-mediated inhibition of protein expression, but also that a functional MRE may reside in 3′-UTR. Yet not all functional MREs are present in the 3′-UTR [14]. Whether this inhibition can also be regulated by another mechanism is yet to be explored.

In human multi-exon genes, 50% ~ 95% of primary transcripts generate isoforms by alternative splicing (AS) or by APA [15-19]. These events greatly increase the functional complexity of the human genome. Some isoforms apparently have active roles in various cell types or tissues [20,21]. Therefore, although alternative splicing is an effective mechanism for generating isoforms, the isoforms may lack functional elements in either the coding or noncoding regions of a primary transcript. A recent systemic study of the Arabidopsis thaliana plant in Yang et al. [22] found a significantly higher frequency of alternative splicing in MREs compared to other regions. However, the relationship between microRNA-mediated inhibition and alternative splicing has not been systematically studied in humans. Moreover, no studies have investigated whether splicing-regulated MRE is highly repressive or functional. Therefore, this study analyzed not only the relationship between alternative splicing and microRNA-mediated protein inhibition, but also the role of splicing-regulated MREs in protein repression.

## Results

### Discovering tissues enriched with specific isoforms derived by alternative splicing

Expressed sequence tag (EST) sequences have proven useful as a reference for identifying alternative splicing in mRNA [23,24]. When sufficient EST sequences are available, the number of EST sequences is approximately proportional to the level of gene expression [25]. Therefore, Legendre *et al*. [10] used the number of EST sequences to determine the differential expression of isoforms contained in EST libraries. They further used this method to show that isoforms may or may not contain conserved regulatory motifs. Since many microRNA recognition elements (MREs) reveal evolutionary conservation [26], MRE-containing or MRE-free transcripts should be detected by a similar approach.

Even all EST sequences used in this study were obtained from non-normalized cDNA libraries, the EST approach suffers from several limitations. For example, not observing an EST sequence does not mean that a gene is not expressed. Thus, all the genes analyzed herein have at least 10 EST sequences. Furthermore, the 3′end of a messenger RNA usually has more EST sequences, so the probabilities obtained by Fisher exact test [10] tended to be underestimated. This bias favours the argument made herein concerning the observation of a differential expression of the counts of an isoform between a given tissue and all the other tissues. Therefore, multiple libraries and additional EST sequences are needed for further discovery of significant MREs. From this point of view, the single cell RNA sequencing approach [27] will be better than pooling the information from different libraries. However, the depth of the public RNA sequencing data is not high enough to do a systematic survey.

**Figure 1:**
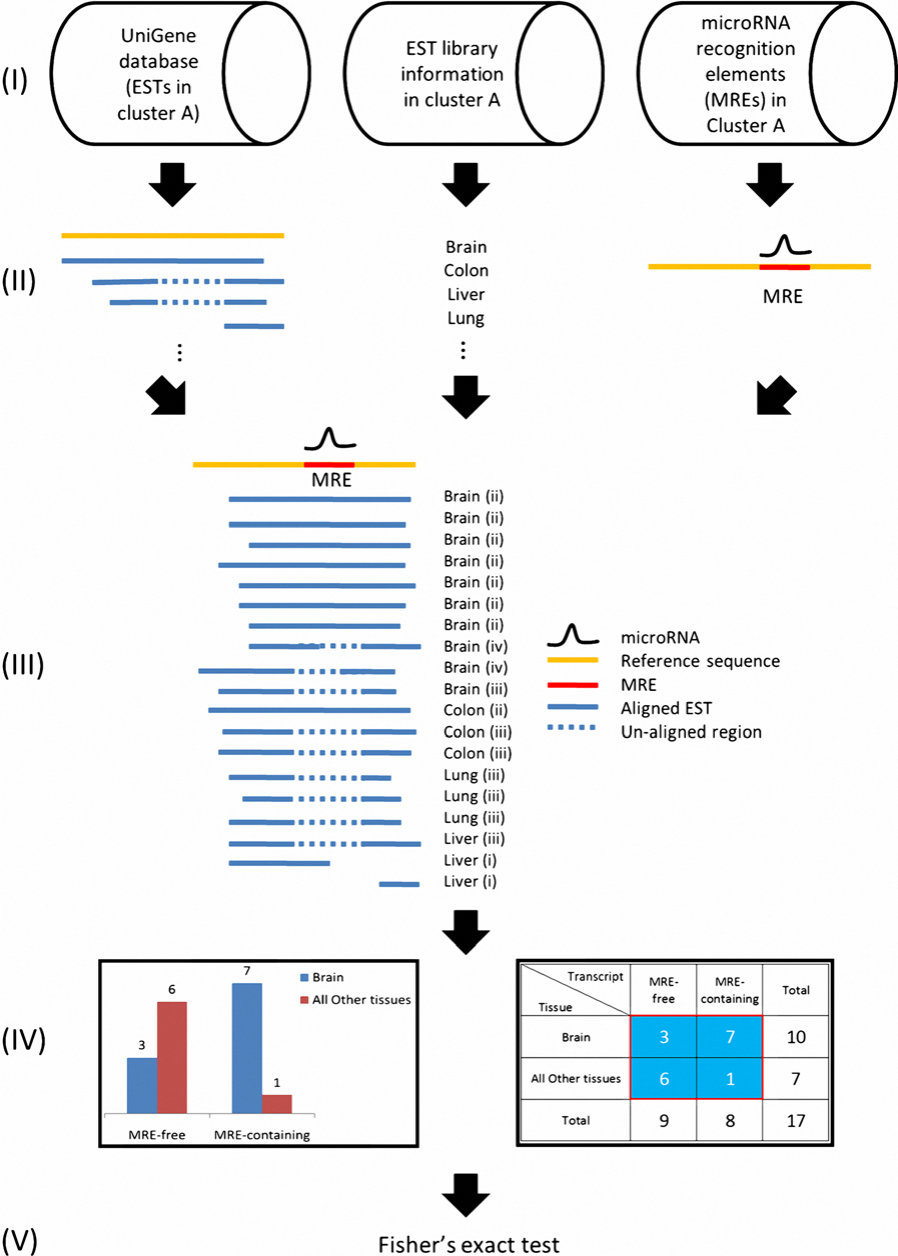
**Method of identifying splicing-regulated MREs in a given tissue.** Step **(I)** is describing the data source used to derive information about alternative splicing events of mRNA, tissue and histology, and MRE. Step **(II)** is schematically depicting the above three data types. Step **(III)** is integrating these three types of information and specifies the EST configuration (see “Methods” for detailed description). Step **(IV)** is establishing the two-way contingency Table used for further analysis. Step **(V)** is performing a statistical test to identify splicing-related MREs that have increased in a given tissue.

Figure 1 shows the example of an MRE present in seven EST sequences but absent from three other EST sequences in brain tissue. However, MRE is present in one EST sequences but absent from six other EST sequences in “all other tissues” (colon, lung, and liver in this example). The two EST sequences from the liver library are excluded since they do not include the entire MRE region. All the aforementioned numbers can be summarized in a two-by-two contingency Table (Figure 1IV and subjected to the Fisher exact test, (Additional file 1: Table S1-1A).

**Figure 2:**
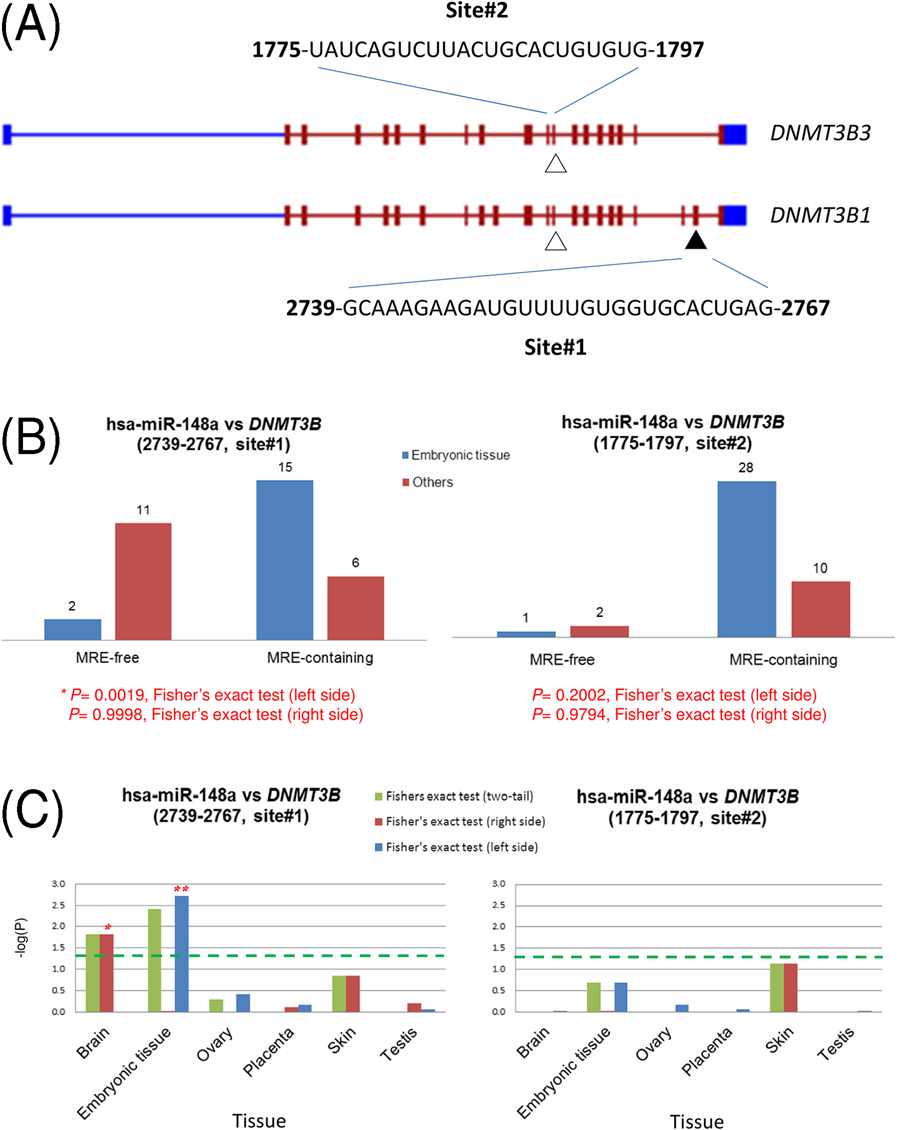
**Splicing-regulated MRE of DNMT3B gene. (A)** Putative hsa-miR-148 recognition sites in human DNMT3B coding region. Symbol “▲” indicated MRE#1 while “△” indicated MRE#2. **(B)** Relative numbers of MRE-containing and MRE-free transcripts from various MRE sites. **(C)** The negative log10 transformed P values in selected tissues. Asterisk represents statistical significance at P < 0.05, determined by Fisher exact test (left or right side).

The DNA methyltransferase 3b (DNMT3B) gene is known to contain two putative MREs that are targeted by the human microRNA hsa-miR-148a [7]. The location of these two MRE sites was found in the coding regions by aligning the reported MRE sequences with the reference sequence of UniGene cluster Hs.713611 (Figure 2A). Transcript DNMT3B3 contains only MRE site#2 (nucleotide 1775–1797), but transcript DNMT3B1 contains both MRE site#1 (nucleotide 2739–2767) and MRE site#2 (Figure 2A and Additional file 1: Figure S1-1). The site#1 MRE-free transcript (*i.e.* DNMT3B3) encodes a protein, which has an in-frame deletion of about 83 amino acids compared to the protein encoded by the site#1 MRE-containing transcript (*i.e.* DNMT3B1). This deletion removes the catalytic site of the methylase activity [28], but it is not clear whether this shorter protein still have the DNA 5-hydroxymethylcytosine dehydroxymethylase activity [29].

The site#1 MRE-free DNMT3B3 transcript may escape translational suppression by hsa-miR-148a, which is expressed in many tissues, such as human embryonic stem cell [30], brain [31], cervix [32] and other tissues [33]. Therefore, the different isoforms transcribed from the DNMT3B gene might be a good starting point to explore the effect of alternative splicing on microRNA-mediated suppression of protein expression.

Since DNMT3B is abundantly expressed in ES cells and early embryos [34], alternative splicing effects can be studied in embryonic tissue. To test whether the highly repressive (repressive ratio ~50%) site#1 MRE [7] is regulated by an alternative splicing event, gene expression was compared between site#1 MRE-containing isoforms and site#1 MRE-free isoforms. Figure 2B shows that, in embryonic tissue, site#1 MRE-containing (*i.e.* DNMT3B1) isoforms are represented by more EST sequences compared to site#1 MRE-free isoforms (*i.e.* DNMT3B3). Left-sided Fisher exact test (see “Methods” for details) showed that the expression in embryonic tissue significantly differed from those of all other tissues (P = 0.0019). In other words, embryonic tissue expressed a higher proportion of site#1 MRE-containing transcript than that in all other tissues and might be more responsive to the hsa-miR-148a-mediated protein repression than that in all other tissues.

In contrast, site#2 MRE-free and site#2 MRE-containing isoforms showed no significant differences between “embryonic tissue” and “all other tissues” based on Fisher exact test. Since the proportion of site#2 MRE-containing transcripts was not significantly changed by alternative splicing event, site#2 MRE was not expected to have a great difference in hsa-miR-148a-mediated protein repression.

### Splicing-regulated site#1 MRE in DNMT3B transcripts is highly repressive

To examine whether alternative splicing may regulate miRNA-mediated protein repression in other tissues, Fisher exact tests were performed in several tissues with sufficient EST sequences. Figure 2C and Additional file 1: Table S1-1B show that the statistically significant P-value obtained by right-side Fisher exact test for brain tissue indicated preferential expression of site#1 MRE-free transcript (*i.e.* DNMT3B3) in the brain. This result in brain tissue was opposite to that in embryonic tissue. The observations based on EST approach were also supported by the single cell RNA sequencing data [27]. For example, the tag counts of those site#1 MRE-containing and site#1 MRE-free isoforms in human embryonic and brain tissues were consistent with the observations by EST approach qualitatively (Additional file 2). Accordingly, alternative splicing is apparently able to regulate protein expression in different tissues.

According to TarBase [35] and miRTarBase [36], hsa-miR-148a has one and seven experimentally verified target messenger RNAs, respectively. In the DNMT3B3 gene, site#1 MRE-free transcript may escape repression of hsa-miR-148a without affecting expression of other targeted genes in the brain. Restated, by changing the properties of a transcript rather than by changing the concentration of a given microRNA, alternative splicing may be an additional regulatory mechanism in protein expression. This additional regulatory mechanism may be useful in fine-tuning the complex gene expression circuit.

The biological importance of microRNA-mediated regulation is typically associated with a highly repressive MRE. Comparisons of microRNA–mediated repression in different mRNA isoforms has been successfully used to associate MRE sites with functions in many studies, such as the prediction of target mRNAs [37-41], the influence of intron retention on human mRNA [42], cellular proliferation and differentiation [12,43], and cancer [44]. The effect of fine-tuning at the splicing level would be negligible if the associated MREs are not highly repressive. Therefore, we predicted that MREs that regulated by splicing are also highly repressive. The DNMT3B analysis in this study showed that only site#1 MRE is highly complementary to hsa-miR-148a and is highly repressive (repressive ratio ~50%) of protein expression [7]. However, site#2 MRE has little no effect (repressive ratio ~10%), which is consistent not only with the above argument, but also with selection pressure in the evolutionary process. That is, if a highly repressive MRE for fine-tuning the microRNA-mediated protein suppression provided a selection advantage, the highly repressive MRE would be preserved in evolution. Therefore, the alternative splicing-modulated protein repression is likely to be associated with highly repressive MRE. In contrast, a weak repressive MRE would confer a smaller selection advantage because of the limited effect of splicing-mediated protein suppression by microRNA. Thus, the weak repressive MRE might no longer be associated with the splicing-level regulation in the evolutionary process.

### Proteomic evidence that highly repressive MREs are generally associated with alternative splicing events

Until now, the association between alternative splicing and highly repressive MRE has been supported only by a single microRNA and a single target messenger RNA. Whether this evidence is applicable to other target messenger RNAs is unclear. Furthermore, gene expression is typically, but not always, proportional to protein expression [45]. Therefore, further studies are needed to determine whether alternative splicing-regulated MREs are generally highly repressive of protein expression.

The global impact of a microRNA, such as miR-124, on the protein expression in the HeLa cell has been measured by stable isotopic labelling with amino acids in cell culture (SILAC) analysis [46], a mass spectrometry technique for using non-radioactive isotopic labelling to detect differences in protein abundance among samples. The SILAC results can also be used to calculate protein expression-based repression. The analysis herein identified 1,544 differentially expressed proteins whose expression resulted from expression of hsa-miR-124 in HeLa cells. However, only 1,486 have symbols in UniGene#230 (and are referred to as the “Proteomics” fraction, Additional file 1: Table S1-2A). Approximately half of these 1,486 proteins were repressed and, of these, approximately half were activated (Additional file 1: Table S1-2B and Text S1-1). These 1,486 proteins identified by proteomic analysis of HeLa cells were used as the starting point in a global analysis.

**Figure 3:**
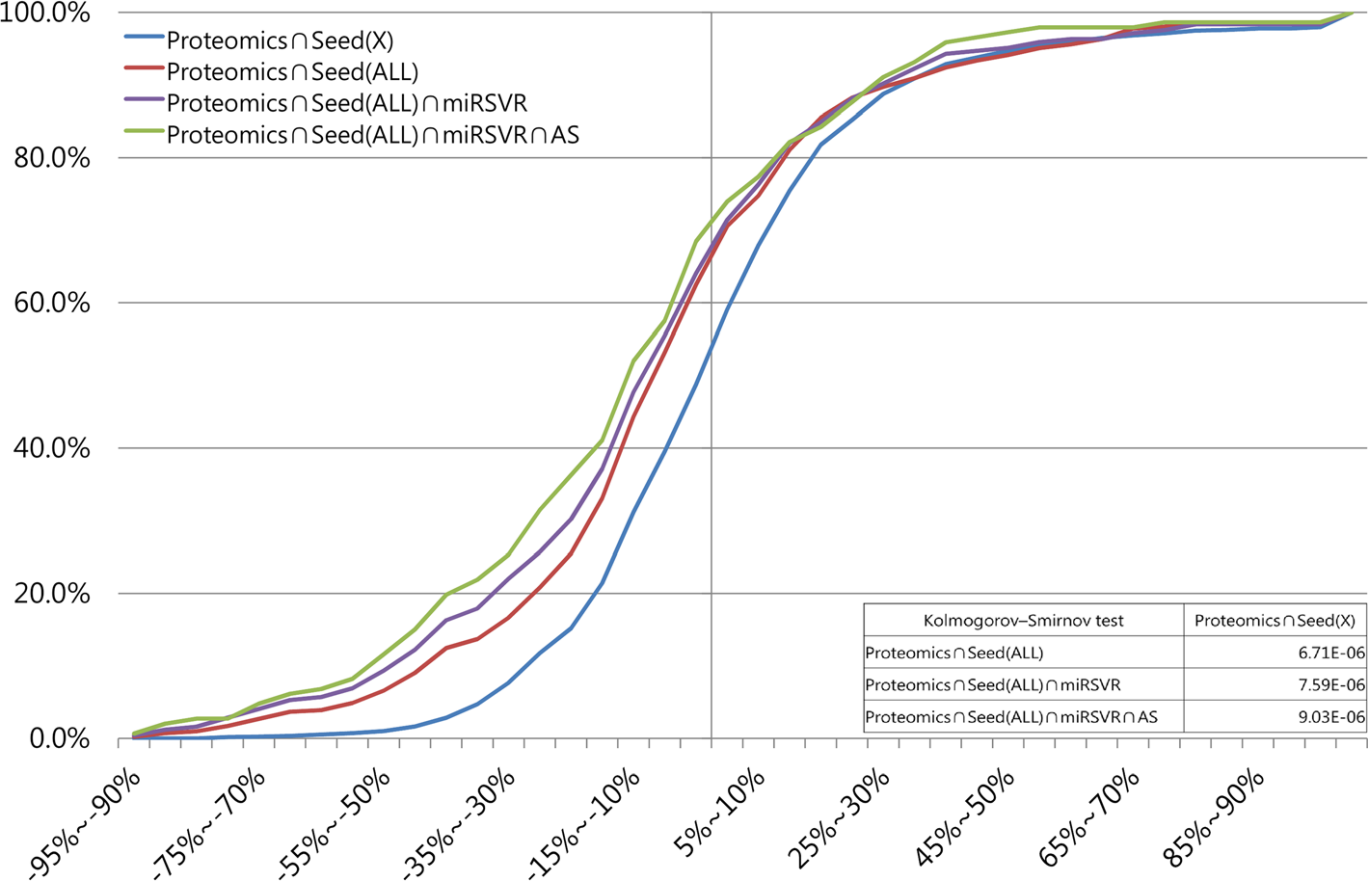
**Cumulative curves representing the cumulate fraction of target proteins that were repressed or activated after transfection of hsa-miR-124 to HeLa cells.** Variation in different states was compared by plotting a cumulative curve [12,19,46,48] of the percentage of proteins in the given range of repression ratios. The x-axis represents the protein output as the percentage change in the expression ratio of HeLa cells. The y-axis represents the cumulative percentage. The text defines the terms proteomics, seed (X), seed (ALL), miRSVR, and AS. All data other than AS information are taken from Baek, *et al*. [46]. A table contained statistic results was also included in the lower right corner.

These proteins include the direct targets of hsa-miR-124 and the downstream proteins of the hsa-miR-124-regulated target genes [47]. Target proteins were distinguished from downstream proteins by labelling the putative target messenger RNA as “seed”, which is a 7-nucleotide feature typically found at the 3′-end of an MRE and complementary to the 5′-end of a microRNA. The presence of a “seed” is thus considered evidence of a candidate MRE. Of these 1,486 proteins, the presence of the seed” region on the transcript indicated that only 408 proteins are directly regulated by hsa-miR-124 (Proteomics∩Seed(ALL), red line in Figure 3). Since the repression is too weak for depiction by histogram, the variation in repression in different states was compared by plotting a cumulative curve [12,19,46,48] of the percentage of proteins in the given range of repression ratios. Figure 3 plots the cumulative curves based on the percentage of counted genes according to the full-length “seed” matches presented in Additional file 1: Table S1-2B and the results of the paired statistical analysis in Additional file 1: Table S1-2C.

The remaining 1,078 seed-free detected proteins were labelled “Proteomics∩Seed(X)” (blue line in Figure 3). The red line showed a significant left shift relative to the blue line (P < 0.001, in a Kolmogorov-Smirnov (KS) test with two independent samples, Additional file 1: Table S1-2C), indicating that repression was greater in the seed-annotated proteins compared to other differentially expressed proteins. However, the presence of a seed usually, but not always correlates with an MRE [37]. Therefore, these “seed”-containing transcripts were further evaluated by the miRSVR algorithm [49] to predict the potential target of hsa-miR-124. These miRSVR-predicted MREs are considered putative MREs. In Figure 3, the cumulative curve of (Proteomics∩Seed(ALL)∩miRSVR) indicated by the purple line is significantly shifted to the left of the blue curve (P < 0.001, KS test, Additional file 1: Table S1-2C), indicating that the target prediction method is better than seed-free detected target messenger RNA. Alternative splicing was then analyzed in target genes (Proteomics∩Seed(ALL)∩miRSVR) encoding differentially expressed proteins. Additional file 1: Table S1-2 presents those proteins that were potentially controlled at the splicing level (Proteomics∩Seed(ALL)∩miRSVR∩AS). The cumulative curve of these splicing-regulated target proteins (green line in Figure 3) exhibited the highest repression, because the green curve shifted further to the left of the blue curve (seed-free target proteins) (P < 0.001, KS test, Additional file 1: Table S1-2C).

In addition to hsa-miR-124, the SILAC analysis [46] was also used to examine the on the global impact of hsa-miR-1 and hsa-miR-181a on protein expression in HeLa cell. This global trend has also been reported in studies of hsa-miR-1 and hsa-miR-181a (Additional file 3), which are consistent with the previous observation on hsa-miR-124. In summary, the ratio of repressive proteins was apparently higher in the alternative splicing-regulated target proteins compared to the remaining differentially expressed proteins. This global analysis of multiple target proteins of a single microRNA supports the association between alternative splicing and highly repressive MRE.

### Splicing-regulated MREs are highly repressive in transcripts that contain multiple MREs

**Figure 4:**
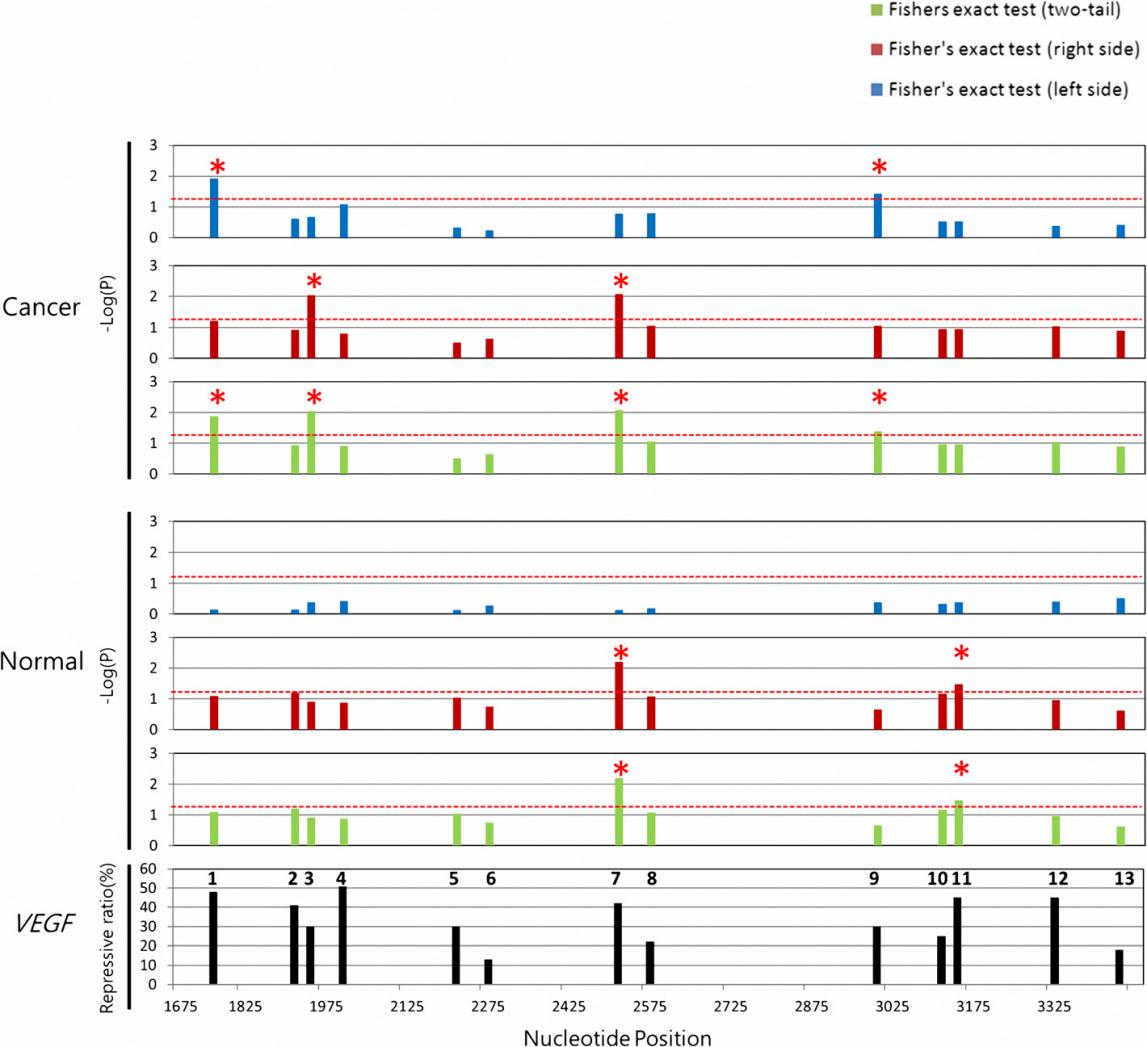
**Comparison of splicing-regulated MRE regions and highly repressive MREs in VEGF gene.** For the alternative splicing analysis of the 13 MRE regions, the top, middle, and bottom panels present the results obtained for cancer tissues, the results obtained for normal tissues, and the repressive ratios reported in the literature, respectively. The negative log10-transformed P-values for the 13 MRE regions (numbered in the bottom panel) are presented in blue, red, and green and correspond to the left-side, right-side, and two-tail Fisher exact tests, respectively. The red star indicates statistical significance at P < 0.05, determined by Fisher exact test. Highly repressive MREs with repression ratios above 30% are found in MRE regions 1, 2, 3, 4, 5, 7, 9, 11 and 12. Putative hsa-miR-148 recognition sites in human DNMT3B coding region. Dotted red line indicates significance region (P < 0.05).

The DNMT3B gene is a good example of the highly repressive splicing-regulated MRE of hsa-miR-148a. The repressive effect is also observed in the target genes of hsa-miR-124 at the protein level. However, many genes are targeted by multiple miRNAs in the cells. For example, approximately 30 putative miRNAs regulate the vascular endothelial growth factor (VEGF) gene [50]. To see the repressive effect and for a fair comparison, different MREs but with at least 90% overlap were grouped into a single “MRE region”. After alignment of the reported MRE regions with UniGene cluster Hs.73793, the splicing-regulated MREs were identified as described above in relation to Figure 1. Thirteen putative splicing-regulated MRE regions were identified (Figure 4) by comparing the locations of MRE regions with splicing sites. The repressive ratio data for these sites were collected in hypoxia-induced CNE cells (a nasopharyngeal carcinoma cell line) by introducing synthetic putative VEGF-regulative microRNA duplexes to this cell line [50] (Figure 4, bottom panel). Of these 13 putative splicing-regulated MRE regions, nine (approximately 70%) were considered highly repressive MRE regions (repressive ratios of +30%): regions 1, 2, 3, 4, 5, 7, 9, 11 and 12 (Table 1, Figure 4, bottom panel).

**Table 1:** Highly splicing-regulated and highly repressive MRE regions in VEGF gene

**Region**	**MRE region**		**microRNA**^**$**^	**Range of repressive ratio (%)**	**Supported tissue from Fisher’s exact test**					
	**Start**	**End**			**Cancer**			**Normal**		
					**Left side**	**Right side**	**Two tail**	**Left side**	**Right side**	**Two tail**
1	1742	1782	hsa-miR-125a(15), hsa-miR-140(48)	15 ~ 48	**Thyroid**		**Thyroid**			
2	1899	1921	hsa-miR-17-5p(35), hsa-miR-20a(39), hsa-miR-20b(41), hsa-miR-106a(21), hsa-miR-106b(35), has-miR-302d(30), hsa-miR-372(30), hsa-miR-520 g(19), hsa-miR-520 h(28)	19 ~ 41						
3	1935	1955	hsa-miR-302d(40)	30		**Ovary**	**Ovary**			
4	1992	2020	hsa-miR-15a(50), hsa-miR-16(51), hsa-miR-195(45)	45 ~ 51						
5	2221	2243	hsa-miR-150(30)	30						
6	2258	2282	hsa-miR-205(13)	13						
7	2508	2554	hsa-miR-15b(25), hsa-miR-107(42), hsa-miR-147(39), hsa-miR-330(25)*	25 ~ 42		**Salivary gland**	**Salivary gland**		**Liver**	**Liver**
8	2567	2601	hsa-miR-34a(15), hsa-miR-34b(8), hsa-miR-373(22), hsa-miR-378(17)	8 ~ 22						
9	3009	3029	hsa-miR-504(30)	30	**Brain**		**Brain**			
10	3134	3160	hsa-miR-383(25)	25						
11	3151	3172	hsa-miR-134(45)	45					**Pancreatic islet**	**Pancreatic islet**
12	3342	3362	hsa-miR-361(45)	45						
13	3466	3486	hsa-miR-29b(18)	18						

^$^ Number in parentheses showed the experimental result of repressive ratio [48]. ^*^ The repressive ratio of hsa-miR-330 is inconsistent from original results.

The cancer (top) and normal (middle) panels in Figure 4 plot the negative log10 transformed P values of all MREs. Four MRE regions showed significant splicing regulation (P < 0.05) in at least one cancer tissue. Two MRE regions appeared to be splicing-regulated in at least one normal tissue. These splicing-regulated MREs were marked with red stars in the two-tail test panels in Figure 4. The normal and cancerous tissues contained five splicing-regulated MRE regions. All five of the splicing-regulated MRE regions (according to Fisher exact test) are also highly repressive MRE regions even though not all highly repressive MREs are regulated by alternative splicing. This implies that analysis of splicing regulation may reveal highly repressive or “true” MREs.

### Approximately one-third of experimentally confirmed MREs are associated with alternative splicing events

Table 1 shows that, in many miRNAs that target VEGF messenger RNA, splicing-regulated MREs were associated with highly repressive MREs. Despite the importance of this observation, this phenomenon is not biologically significant if splicing-regulated MREs are special cases. Therefore, alternative splicing analysis was repeated in experimentally supported human targets based on the reporter assay results [35]. Random MREs were also analyzed to evaluate background effects. Notably, the true MRE was positively validated by the algorithm, the false MRE was negatively validated by the algorithm [35], and the random MRE was simply randomly selected from reference sequences. If our observation of DNMT3B is widely applicable, then the opportunity to observed splicing-regulated MRE would be greater for the experimentally verified MREs than for the false or random MREs.

The sequences of translationally repressed human MREs and false MREs were retrieved from both miRTarBase release 2.5 [36] and TarBase v.4.0 [35] and aligned to the corresponding reference sequences in UniGene database (see Methods). Only MREs with at least ten EST sequences were included in further analyses. The analysis included 256 unique MRE sites obtained from miRTarBase and 72 unique MRE sites and 14 unique false sites obtained from TarBase. From the 3′UTR and full-length sequences, 170,000 and 431,500 distinct random MREs were generated, respectively.

**Figure 5:**
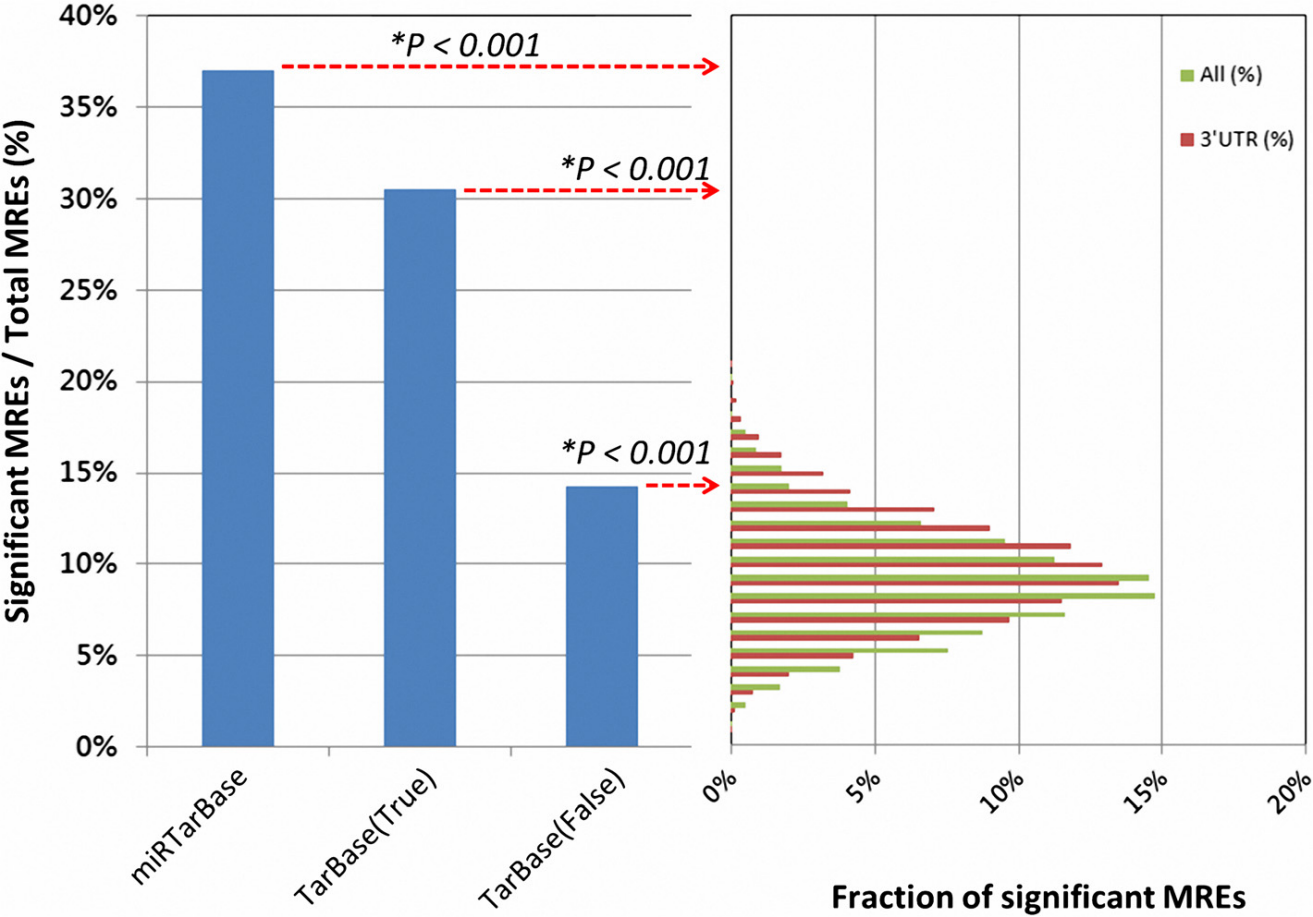
**Comparison of predicted splicing-regulated MREs from true, false, and randomly selected MREs.** The left panel shows the fractions of splicing-regulated MREs contained in miRTarBase [36] and TarBase [35]. True MRE is the algorithm-predicted MRE with positive validation, and false MRE is the algorithm-predicted MRE with negative validation defined from TarBase. The right panel shows a histogram of splicing regulation predicted in MREs randomly selected from reference sequences. Data for random MREs were averages over 4,315 or 1,700 randomly selected MRE sets (100 random MREs/set), which were sampled from full-length (green) and 3′-UTR (red) sequences, respectively. P-value was computed by one-sample Wilcoxon signed-rank test.

The number of unique MRE sites was determined by applying the following two rules. First, an MRE targeted by multiple microRNAs (e.g., hsa-miR-20a and hsa-miR-17 both targeting the same MRE in the E2F1 gene) was considered a single site. Second, significant tissue types (Additional file 1: Figure S1-2) identified in the alternative splicing analysis of a given MRE were considered OR significant tissue types (Additional file 1: Figure S1-2) identified in the alternative splicing analysis of a given MRE were defined as splicing-regulated. Alternative splicing in at least one tissue was identified in 95 out of 256 (37.1%) MRE sites in miRTarBase and in 22 out of 72 (30.6%) MRE sites in TarBase (Figure 5). These percentages were 2.2 and 2.6 times those of the false MRE sites in TarBase and miRTarBase, respectively. The percentages of both the true and false MRE sites significantly differed (average, 9.7%) from those of the random MRE sites selected from 3′ UTR or full-length sequence (P < 0.001, one-sample Wilcoxon signed-rank test). A possible explanation for the difference is that negative validation of the algorithm-predicted MRE has been reported for only one or a few tissues, which may significantly differ from other tissues in the EST libraries. The percentage of significant randomly selected MREs in this analysis (9.7%) also differed from those in Additional file 1: Figure S1-2B (2.8%) because the latter treated MREs as independent in different tissues.

**Figure 6:**
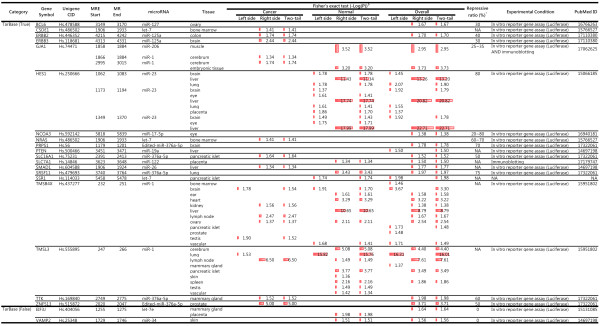
**Predicted highly repressive MREs from TarBase v.4.0. **^$^ Red bar indicate the level of significance; ^*^ Listed numbers were experimental repression ratios obtained manually from full-text cited by TarBase.

The experimental results show that approximately one-third of the true MREs were associated with AS events. Additionally, reported repressive ratios exceeded 30% in almost all proteins in Figure 6. Restated, the association between splicing-regulated MREs and highly repressive MREs is again supported by other microRNAs and their target messenger RNAs.

## Discussion

This study showed that splicing-level regulation is a fine-tuning mechanism in the expressions of specific proteins. The fine-tuning process described above enables regulation of a single protein in the presence of all the other target proteins of a given microRNA. Briefly, specific examples of this process were extended to all analysable human targets in TarBase and miRTarBase. The microRNA-mediated regulation is quite complex. For example, a single microRNA may have multiple target genes, and a messenger RNA may be targeted by multiple microRNAs. Alternative splicing enables independent regulation of protein expression in a single gene. An MRE-containing isoform can be co-regulated with other target genes, but an MRE-free isoform may escape regulation of the matching microRNA.

Additionally, splicing-regulated MREs established here were highly repressive (repressive ratios +30%). This highly repressive character may enhance the effectiveness of alternative splicing. The fine-tuning process may exert a positive selection pressure in evolution. This effect may explain why almost all observed splicing-regulated MREs were highly repressive. However, not all genes require fine-tuning. Therefore, highly repressive MREs may not necessarily be regulated by alternative splicing. For example, the highly repressive (repressive ratio 75%) hsa-miR-137-specific MRE in the CDC42 gene [22] is not associated with alternative splicing (data not shown).

The discussed fine-tuning mechanism also suggests a means for cancer cells to escape regulation in a normal cell. As presented in Figure 6, enrichment of splicing-regulated MRE regions was significantly increased in cancer cells according to right-sided Fisher exact test. This right-side significance indicates an increased amount of an MRE-free transcript in cancer cells. Restated, genes such as CSDE1, ERBB2, ERBB3, GJA1, N-RAS, SLC16A, SMAD1, TMSB4X, TMSL3, TTK, and ZNF513 were up-regulated in cancer cells. This result is consistent with the notion that a loss of repressive elements located in 3′ untranslated regions (3′UTRs) by alternative cleavage and polyadenylation (APA) was associated with oncogene activation [13] and cellular activation [12]. Thus, the above association is not only a common phenomenon, it also has important biological implications.

Regarding applications, identifying functional MREs is critical to determining the biological function of microRNAs. However, measuring the repressive ratio at an MRE site is laborious. Although the prediction algorithm considered many factors, the false positive rate was unacceptably high [3,50,51]. Therefore, predicting the functional MREs of a given microRNA remains challenging [52,53]. Our experimental results suggest that alternative splicing can also be used to filter and identify functional MRE sites from a list of putative sites predicted by microRNA target identification algorithms. The false-negative rate of the suggested method of identifying novel targets of miRNAs is high since approximately two-thirds of the true MREs were unassociated with AS events. Although this method is not comprehensive, the false positive rate is low. More importantly, most of these splicing-regulated MREs are highly repressive MREs, which have functional implications.

## Conclusions

In conclusion, the discovery of splicing-regulated MREs reveals an important fine-tuning mechanism in complex microRNA-mediated regulation. This phenomenon is widely observed in human cells and is accompanied by the strong repression of protein expression. This novel and biologically significant observation is useful for identifying MREs, which may have important biological functions.

## Methods

### EST data source and prediction of spliced sites

Publically available Expressed Sequence Tag (EST) sequences were used to discover isoforms of messenger RNA [24,54,55]. Alternatively spliced isoforms can be identified by aligning EST sequences with reference messenger RNA sequences. Tissues that express a particular EST sequence can also be annotated with NCBI information. The MRE-containing or MRE-free isoforms can then be identified by comparing the locations of functional MREs with alternative splicing (AS) sites. The UniGene (Human, Build #230) database was used to discover the alternative splicing sites by aligning each EST sequence with the reference sequence, which was the longest high-quality sequence in the NCBI (file “Hs.seq.uniq”). Alignment was performed in sim4 using the default parameters [56] as described by Huang, *et al*. [24]. The relationship between a putative splicing site and an MRE site (see below for details) was determined by comparing the locations of the above two features.

### Alignment of experimentally verified and predicted MREs with the reference sequence

The known microRNA genes were obtained from miRBase (version 17.0) [41] and the experimentally verified MREs were downloaded or retrieved from TarBase (version 4.0) [35] and miRTarBase (release 2.5) [36]. For each predicted MRE, information obtained from microRNA.org (August, 2010 Release) [57] included potential binding sites identified by using the mirSVR [49] and miRanda [58] algorithms for a given microRNA. The MRE positions were then identified by using the BLASTN algorithm [59] to match each MRE with the reference sequences and then retaining only those with perfect matches over their full length.

### Configuration of EST sequence and Fisher exact test

As in step (III) of Figure 1, information about alternative splicing, tissue, histology (normal vs. cancer), and MRE were integrated. Tissue and histology information were obtained from the EST library established by the Cancer Genome Anatomy Project [60,61]. After the splicing and MRE information were integrated, each EST sequence had five possible configurations in relation to a specified MRE sequence: (i) partial or no overlap with MRE; (ii) absence of MRE in a putative exon; (iii) presence of MRE in a putative intron; (iv) partial MRE at the exon-intron junction, and (v) other. Each EST sequence was assigned a configuration number in this step (*e.g.*, see the parenthesis after a given tissue name in step (III) of Figure 1) and was classified accordingly. Each configuration had a unique biological meaning. In configuration (i), the EST sequence was unrelated to alternative splicing. In configuration (ii), the EST sequence was in an MRE-containing isoform. In configuration (iii), the EST sequence was in an MRE-free isoform. In configuration (iv), the isoform contained only a partial, and potentially non-functional, MRE. Therefore, the isoform was classified as an MRE-free isoform.

The EST sequences were grouped according to whether they were MRE-containing or MRE-free isoforms in various tissues and histologies. In step (IV) (Figure 1), a two-way contingency table was established to determine whether the counts of MRE-free or MRE-containing isoforms were over-represented in a given tissue, in relation to “all other tissues”. The statistical analyses were limited to tissues with at least ten EST sequences. The P-value was computed by Fisher exact test (Figure 1 and Additional file 1: Table S1-1A) to identify non-random associations between two categorical variables. The null hypothesis was that, in the absence of natural selection, the proportion of (MRE-free isoforms/MRE-containing isoforms) in a given tissue is identical to that in all other tissues. Therefore, the hypergeometric distribution was used to calculate the probabilities of the observed data and all data sets with more extreme deviations. When a given tissue showed enriched MRE-containing isoforms, the left-side P-value was significant in one-tailed Fisher exact test. In contrast, when a given tissue showed increased MRE-free isoforms, the right side P-value was significant in a one-tailed Fisher exact test.

### Preparing random sequences for Wilcoxon signed-rank test

To observe the background effect of the configuration in Figure 1, 170,000 and 431,500 different MREs were randomly generated from 3′UTR and full-length sequences, respectively. The numbers of random MREs sufficed for the purposes of this study since 2.8% of 100,000 random MREs were putative splicing-regulated MREs (P < =0.05, see red line in Additional file 1: Figure S1-2B indicating accumulated probability of all MREs randomly selected for statistical analysis). These reference sequences were randomly selected from the UniGene database for use as source sequences. The sequence source, the starting position of a random sequence, and the length (18 ~ 22 nucleotides) of a random sequence were all randomized. Once the starting site had been determined, an MRE sequence was randomly generated from the source sequence under the constraint of MRE length. Therefore, all selected MREs were in the source sequences and were included in the isoform enrichment analysis described above. Since the amounts of splicing-regulated random MREs were not normally distributed among these two sets (Figure 5, right panel), one-sample Wilcoxon signed-rank test was used to differentiate between true MREs and randomly generated MREs.

## Competing interests

The authors declare that they have no competing interests.

## Authors’ contributions

CTW designed and performed the major analysis and wrote the first draft of this manuscript; CYC provided the alternative splicing data; HCC performed the single-cell level mRNA-seq analysis; UCY reorganized and revised the manuscript. All authors read and approved the final manuscript.

## Supplementary Material

Additional file 1 Supplementary texts, figures and Tables. Click here for file

Additional file 2 Single-cell level mRNA-seq analysis of human embryonic and brain tissue. Click here for file

Additional file 3 Global trend of target proteins that were repressed or activated after transfection of hsa-miR-1 or hsa-miR-181a to HeLa cells. Click here for file
