# Supplementary material for: Fine-tuning of microRNA-mediated repression of mRNA by splicing-regulated and highly repressive microRNA recognition element
Source: BMC Genomics. 2013 Jul 3;14:438. doi: 10.1186/1471-2164-14-438 (PMC3708814; doi:10.1186/1471-2164-14-438)
Supplement: Additional file 1 — Supplementary texts, figures and Tables. [file 1471-2164-14-438-S1.doc]

# Fine-tuning of microRNA-mediated repression of mRNA by splicing-regulated and highly repressive microRNA recognition element

Cheng-Tao Wu, Chien-Ying Chiou, Ho-Chen Chiu and Ueng-Cheng Yang

# Additional files

### Additional file 1– Supplementary texts, figures and Tables

1. Text S1-1: Supplementary text for proteomics analysis
2. Text S1-2: Supplementary Table and figure legends
3. Supplementary figures

Figure S1-1

Figure S1-2

Figure S1-3

1. Supplementary figures

Table S1-1

Table S1-2

**Additional file 1**

**Text S1-1**

**Supplementary text for proteomics analysis**

As described in supplementary Table S1-2(A), Baek *et al.* identified 1,544 proteins by SILAC analysis following expression of miR-124 in the cells; 1,486 have symbols in UniGene#230 (and are referred to as the “Proteomics” fraction). About half exhibited a repressive effect and the other half exhibited an activation effect. The identities of 408, 12, 215 and 216 of these 1,486 proteins were further supported by the “seed” matches in the corresponding regions of “full length”, “5’UTR”, “ORF” or “3’UTR” of a gene (these fractions are called“Proteomics∩Seed(ALL)”, “Proteomics∩Seed(5'UTR)”, “Proteomics∩Seed(ORF)”, “Proteomics∩Seed(3'UTR)”), respectively. Restated, the messenger RNAs that encode the 408 proteins contain the first two to seven nucleotides that are complementary to miR-124. The rest of the 1,078 (1,486 – 408) seed-free, proteins are called “Proteomics∩Seed(X)” fraction. To understand the significance of the increase in the amount of highly repressive MREs, the predictions in which the potential MREs are identified by the miRSVR and miRanda algorithms for a given microRNA from the microRNA.org are exploited (August 2010 Release) . Restated, each fraction was further combined with the target prediction result of miR-124 from microRNA.org. For example, only 544 out of 1,486 proteins were predicted to undergo at least one miR-124:MRE interaction. (This fraction is called “Proteomics∩miRSVR”). For another example, in the fraction “Proteomics∩Seed(3'UTR)”, only 190 proteins were predicted to exhibit at least one miR-124:MRE interaction. (This fraction is called “Proteomics∩Seed(3'UTR)∩miRSVR”). When this fraction (“Proteomics∩Seed(3'UTR)∩miRSVR”) is combined with the method proposed herein, 119 out of these 216 proteins were affected by alternative splicing. (This fraction is called “Proteomics∩Seed(3'UTR)∩miRSVR∩AS”). By comparing the ability of the miR-124:MRE interaction to repress protein expression, the increase in the amount of highly repressive MRE in these fractions. In Fig. 3, the cumulative curves were plotted on the basis of the percentage of counted genes that were presented in supplementary Table S1-2(B) and the results of the paired statistical analysis in supplementary Table S1-2(C).

As expected, the “Proteomics∩miRSVR∩AS” fraction differs significantly from the “Proteomics” fraction (Fig. S3(A), P=0.029, in a Kolmogorov-Smirnov test with two independent samples). Additionally, the “Proteomics∩Seed(3'UTR)∩miRSVR∩AS” fraction differs significantly from the “Proteomics∩Seed(X)” fraction (P<0.001). However, the “Proteomics∩Seed(3'UTR)∩miRSVR∩AS” fraction does not differ significantly from the “Proteomics∩Seed(3'UTR)∩miRSVR” faction (Fig. S1-3(C)). However, the number of proteins was reduced from 190 to 119 (37.3%) between these fractions. The four curves reveal that the “Proteomics∩Seed(3'UTR)∩miRSVR∩AS” fraction has more highly repressive proteins with over 30% repression than the “Proteomics∩Seed(X)” fraction. In particular, the method herein predicted all of the top four down-regulated proteins, indicating that our hypothesis was also verified at the protein level. Similar results are obtained by comparing “ORF”-related fractions (Fig. S1-3(B)).

**Text S1-2**

**Supplementary table and figure legends**

**Table S1-1.** (A) Frequency data associated with transcript status and tissue information, presented in a 2x2 contingency table. (B) Statistical analysis of AS-affected MREs from *DNMT3B* (UniGene cluster Hs.713611).

**Table S1-2.** (A) Definition and number of detected targets of various fractions. (B) Numbers of genes with decreased or increased level caused by the expression of hsa-miR-124 in HeLa cells relative to mock control from various fractions. (C) Paired statistical analysis of various fractions. A P-value of under 0.05 is considered significant and indicated in red.

**Figure S1-1.** Verified miR-148a sites in DNMT3B1 coding region. (A) Repressive ratio of target site#1 and #2; (B) Putative alternative splicing events in PALSDB (Putative Alternative Splicing Database) . Strong AS event is evident at site#1.

**Figure S1-2.** (A) Flowchart for estimating the background using randomly selected MREs. (B) Probability distribution of randomly selected MREs in (A). MRE from different tissues are treated independently. **Blue line** is the fraction and **red line** is the cumulative fraction of random MREs. The P-values are indicated on the x axis. Dotted line (in **orange**) indicates that P-value of 95% of the 100,000 random MREs is larger than 0.079. Only 2.8 % of 100,000 random MREs were putative splicing-regulated MREs (P<=0.05, dotted line (in **green**)).

**Figure S1-3.** hsa-miR-124 transfection significantly increases amounts of gene with AS event and seed-supported MREs. Cumulative curves present the cumulated fraction of target proteins that were repressed or activated after transfection of hsa-miR-124 to HeLa cells. The x-axis represents the protein output as a percentage of expression ratio change of the control HeLa cells. The y-axis represents the cumulative percentage. The text on the upper left corner presents the meaning of each curve. Proteomics means all the proteins that are differentially expressed upon the treatment with miR-124. Seed-free target genes are represented by seed(X). Target genes that have seeds found in the 3’UTR is represented by Seed(3’UTR). Target genes that have seeds found in the ORF is represented by Seed(ORF). Target genes that have seeds found in the full length is represented by Seed(ALL). The seeds detected by the miRSVR program is represented by miRSVR. The target proteins that are regulated by alternative splicing is represented by AS. All the data except for the AS information are taken from Baek, et al. (2008). (A) Comparison of all proteomics output with miRSVR with AS-supported proteomics output; (B) comparison of seed-free proteomics output with seed-in-ORF, miRSVR and AS-supported proteomics output; (C) comparison of seed-free proteomics output with seed-in 3’UTR, miRSVR and AS-supported proteomics output; (D) Comparison of seed-free proteomics output with seed-in-3’UTR or seed-in-ORF or seed-supported proteomics output, miRSVR and AS-supported proteomics output.

**Supplementary figures**


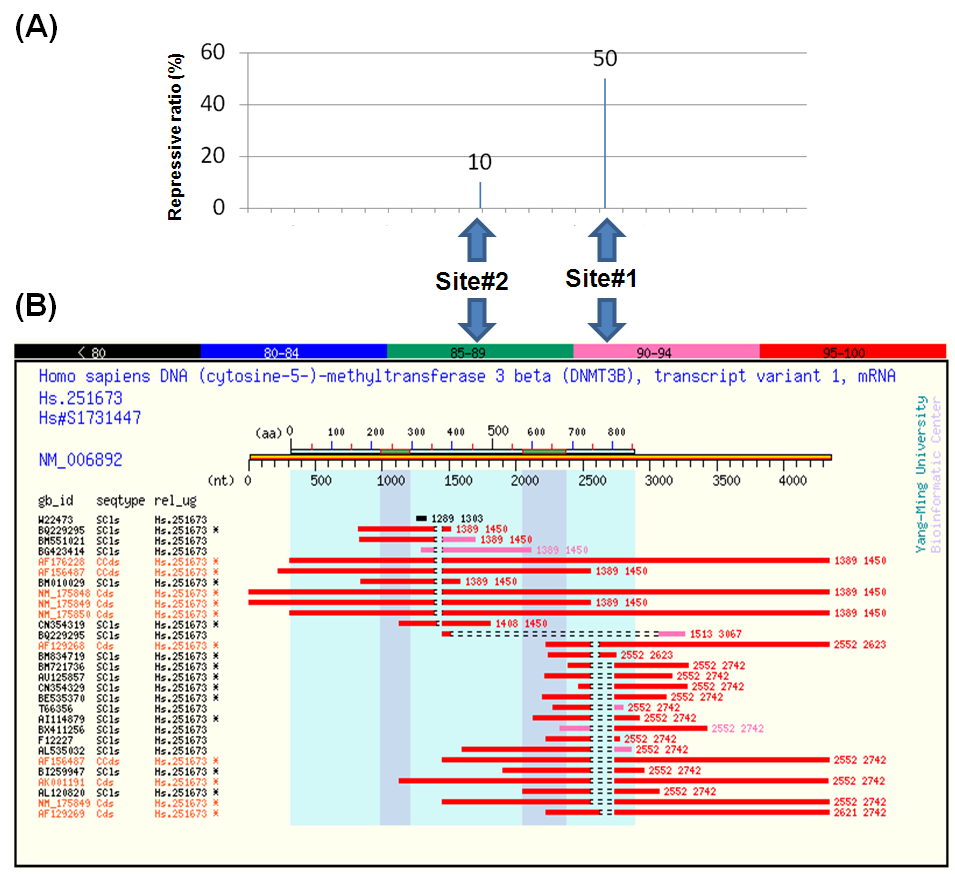


Figure S1-1


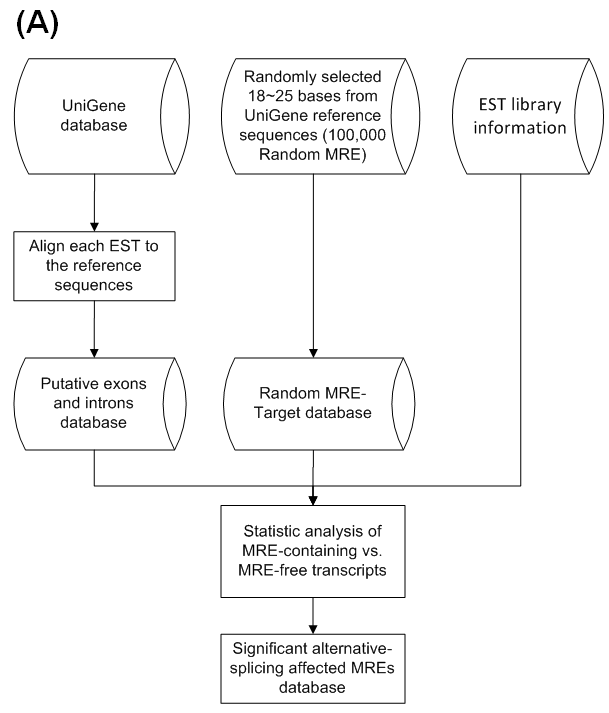

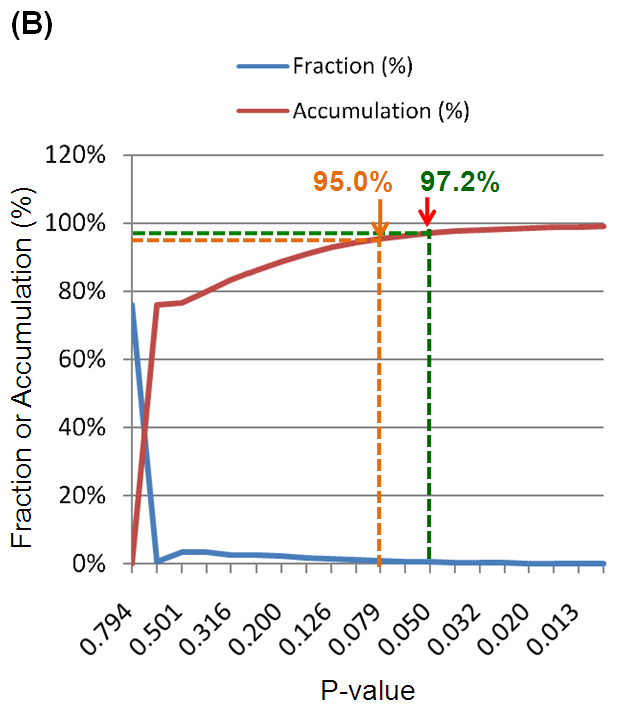


Figure S1-2

(A)


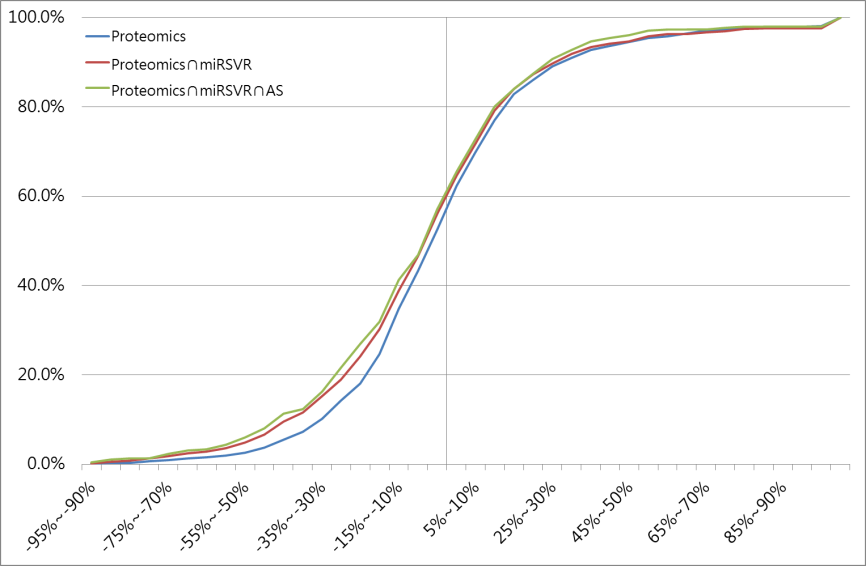


(B)


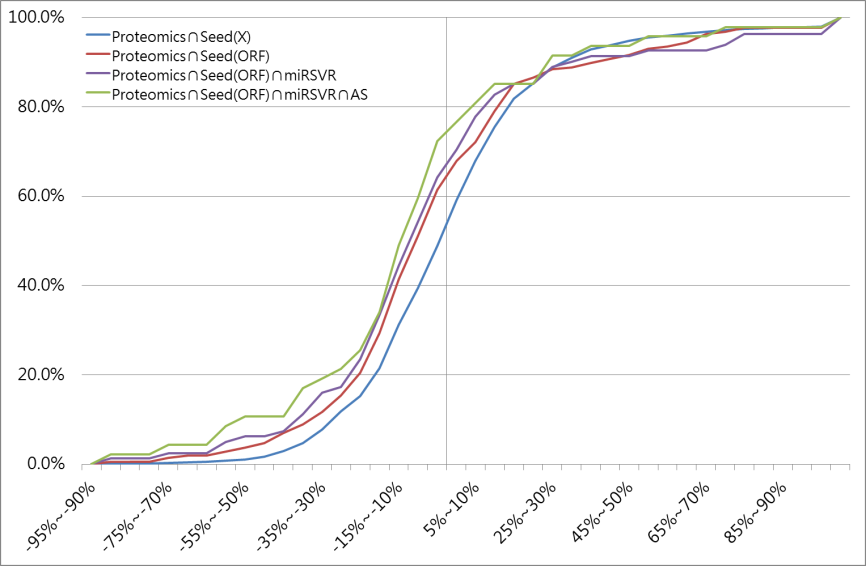


(C)


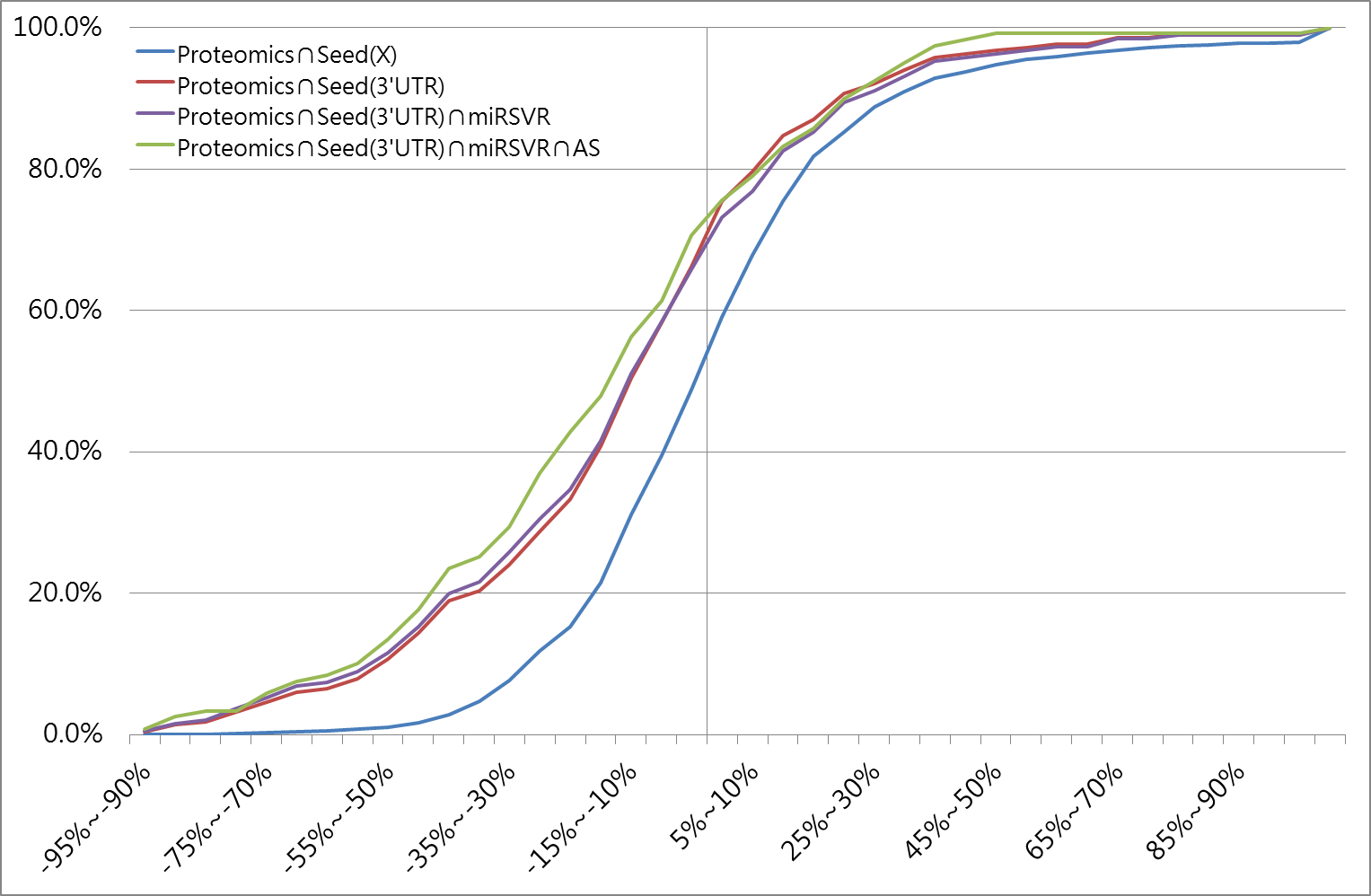


(D)


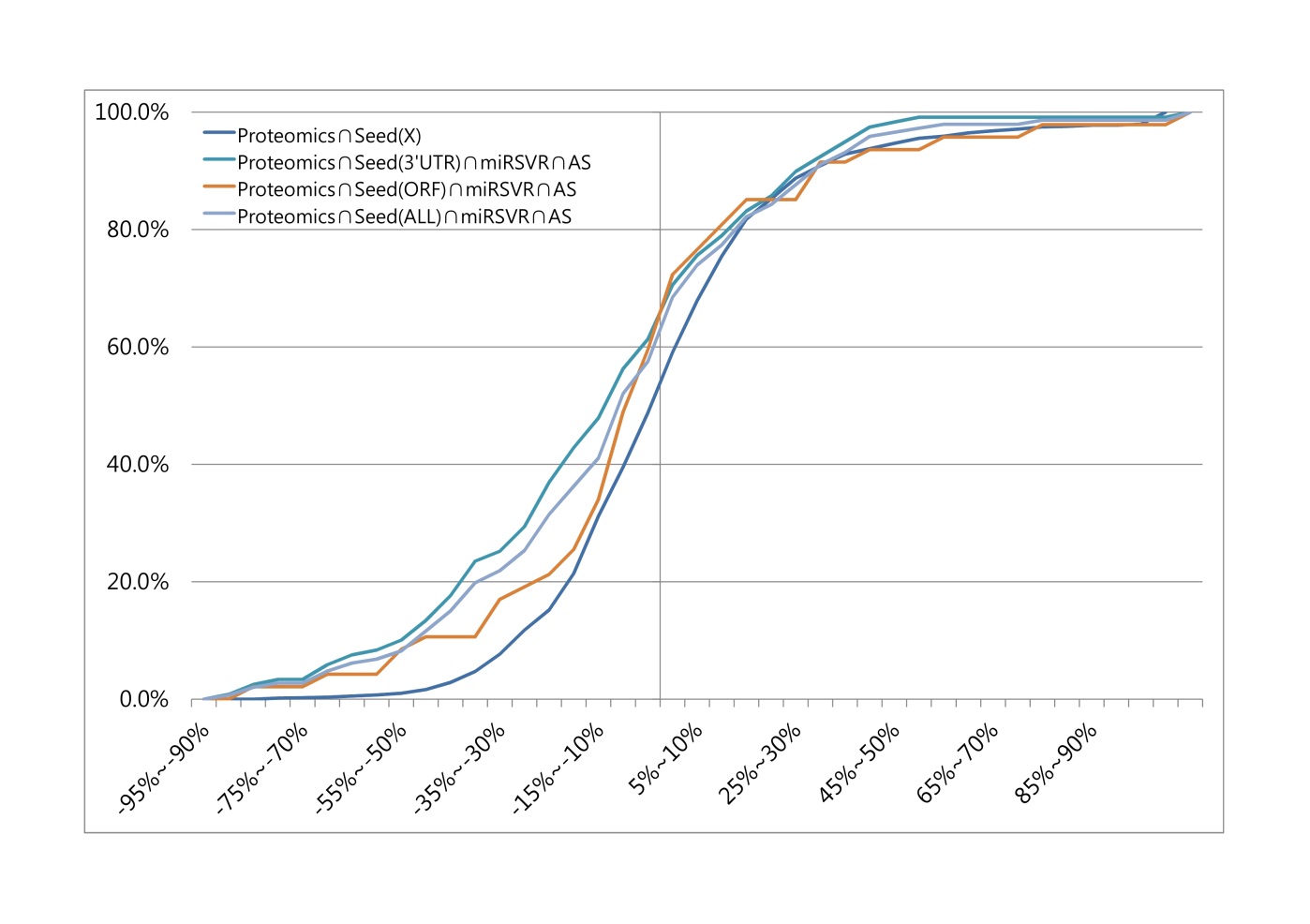


*Please refer to Table S2(B) to see the result of paired statistical analysis (K-S test).

Figure S1-3

**Supplementary tables**

Table S1-1

Table S1-2

* The statistical analysis is not performed due to small number of detected targets from 5’UTR.

Reference

1. Betel D, Koppal A, Agius P, Sander C, Leslie C: **Comprehensive modeling of microRNA targets predicts functional non-conserved and non-canonical sites**. *Genome Biol* 2010, **11**(8):R90.

2. John B, Enright AJ, Aravin A, Tuschl T, Sander C, Marks DS: **Human MicroRNA targets**. *PLoS Biol* 2004, **2**(11):e363.

3. Betel D, Wilson M, Gabow A, Marks DS, Sander C: **The microRNA.org resource: targets and expression**. *Nucleic Acids Res* 2008, **36**(Database issue):D149-153.

4. Huang YH, Chen YT, Lai JJ, Yang ST, Yang UC: **PALS db: Putative Alternative Splicing database**. *Nucleic Acids Res* 2002, **30**(1):186-190.
