# Supplementary material for: Fine-tuning of microRNA-mediated repression of mRNA by splicing-regulated and highly repressive microRNA recognition element
Source: BMC Genomics. 2013 Jul 3;14:438. doi: 10.1186/1471-2164-14-438 (PMC3708814; doi:10.1186/1471-2164-14-438)
Supplement: Additional file 2 — Single-cell level mRNA-seq analysis of human embryonic and brain tissue. [file 1471-2164-14-438-S2.doc]

**Additional file 2**

**Single-cell level mRNA-seq analysis of human embryonic and brain tissue**

Recently, a method for single-cell mRNA-Seq (Smart-Seq) has been introduced that quantifies transcripts through full-length . To evaluate the results from EST analysis (see Methods) by mRNA-seq, ten selected libraries (GSE38495) from this publication were obtained from Gene Expression Omnibus (**Table S2-1**). Alignments were performed by using GSNAP , an alignment program based on GMAP with “--use-splicing” and “--novelsplicing” parameters for aligning short reads from mRNA-seq to human reference genome sequence (hg19). The results were converted into bigWig format (**Table S2-1**) with Rsamtools followed by rtracklayer using R-2.15.3 and visualized by Ensembl (**Figure S2-1**).

For DNMT3B gene (23 exons in total) we extracted the reads mapped by GSNAP overlapping the gene region using SAMTools and performed MRE-free and MRE-containing reads-counting of MRE#1(**Table S2-2A**) and MRE#2(**Table S2-2B**) with in-house scripts. Transcript DNMT3B1 is composed of all 23 exons, while MRE#1 is located in exon 22 and MRE#2 in exon 14. In addition to MRE regions analysis, because in DNMT3B3 exons 10, 21 and 22 are absent, reads in exon20-exon23 junction were also calculated for (exon21-exon22 containing) / (exon21-exon22 free) comparison. Table 2(A) shows that, in embryonic tissue (hESC), site#1 MRE-containing isoforms were represented by more reads compared to site#1 MRE-free isoforms (MRE-containing: MRE-free = 12~45 reads : 0 reads). On the other hand, MRE-free isoforms were represented by more reads in universal human reference RNA (MRE-containing: MRE-free = 7~10 reads : 0 reads). Interestingly, high percentage of MRE-free reads (50%~100%) were in exon20-exon23 junction.

**Supplementary table and figure legends**

**Table S2-1.** List and description of selected single-cell levels mRNA-seq libraries.

**Table S2-2.** Mapped reads extracted by GSNAP overlapping DNMT3B gene region using SAMTools and performed MRE-free and MRE-containing reads-counting of (A) MRE#1 and (B) MRE#2.

**Fig. S2-1.** BigWig format of the selected 8 libraries can be visualized by Ensembl.

**Table S2-1**.

| Sample | Sample ID | Description | Alignment |
| --- | --- | --- | --- |
| GSM967575 | SRR522148 | Universal human reference RNA s_8_HCT21008-UHR-1ng | SRR522148.bigWig |
| GSM967572 | SRR522145 | Universal human reference RNA s_7_HCT21007-UHR-1ng | SRR522145.bigWig |
| GSM967509 | SRR522082 | cell2_hESC | SRR522082.bigWig |
| GSM967508 | SRR522081 | cell1_hESC | SRR522081.bigWig |
| GSM967504 | SRR522077 | Human brain s_6_HCT21006-BR-1ng | SRR522077.bigWig |
| GSM967502 | SRR522075 | Human brain s_5_HCT21005-BR-1ng | SRR522075.bigWig |
| GSM967491 | SRR522064 | Human brain HCT20421-BR1ng | SRR522064.bigWig |
| GSM967490 | SRR522063 | Human brain HCT20413-BR1ng | SRR522063.bigWig |
| GSM967495 | SRR522068 | Human brain s_1_HCT21001-BR-10ng | SRR522068,bigwig |
| GSM967498 | SRR522071 | Human brain s_2_HCT21002-BR-10ng | SRR522071.bigWig |

**Figure S2-1.**


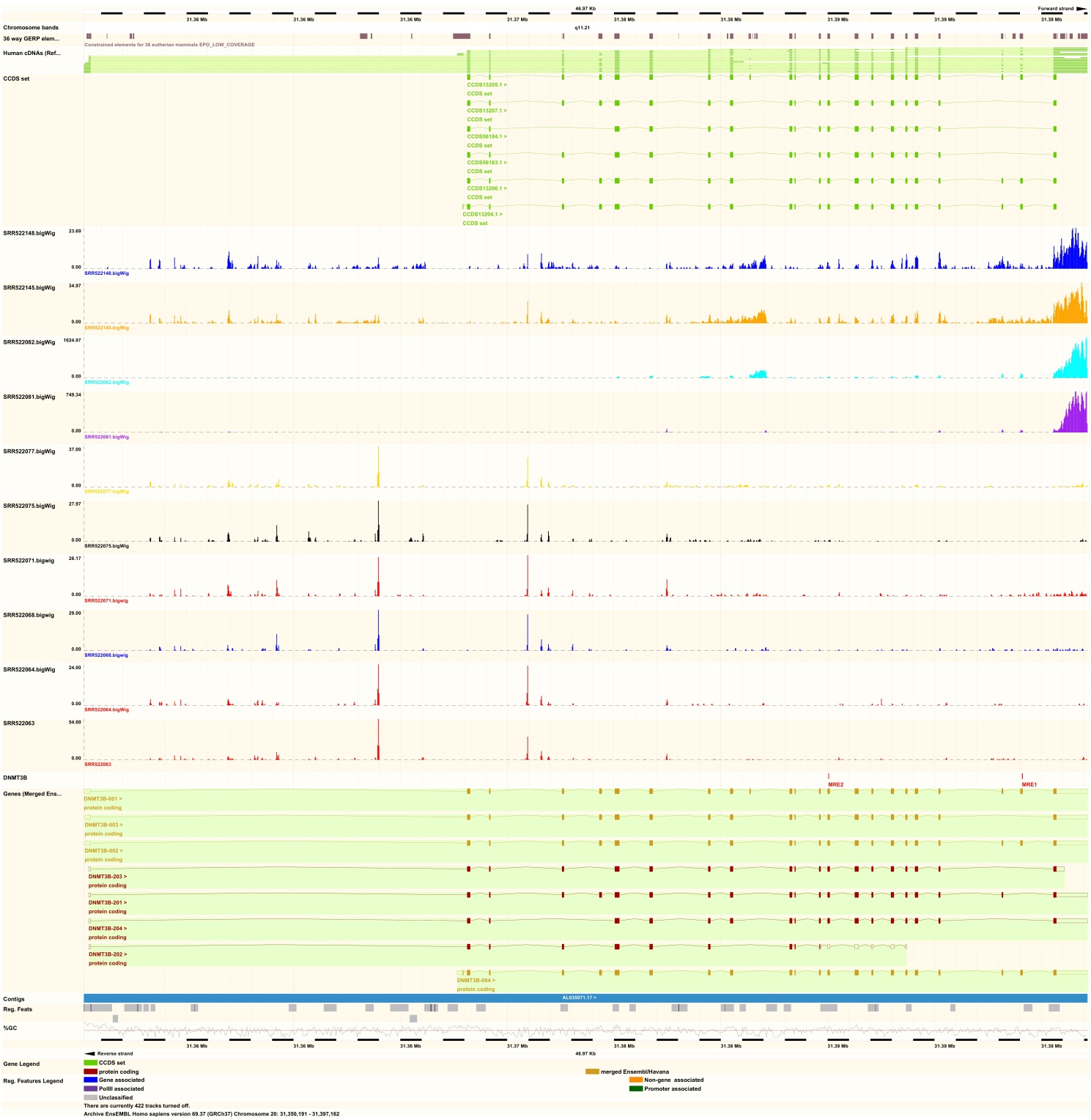


**Table S2-2. (A)**

| MRE#1: 20:31394097-31394125 | | | | |
| --- | --- | --- | --- | --- |
| Description | Sample ID | MRE-containing | MRE-free | Reads in exon20-exon23 junction |
| Universal human reference RNA s_8_HCT21008-UHR-1ng | SRR522148 | 0 | 7 | 7 |
| Universal human reference RNA s_7_HCT21007-UHR-1ng | SRR522145 | 0 | 10 | 5 |
| cell2_hESC | SRR522082 | 45 | 0 | 0 |
| cell1_hESC | SRR522081 | 12 | 0 | 0 |
| Human brain s_6_HCT21006-BR-1ng | SRR522077 | 0 | 0 | 0 |
| Human brain s_5_HCT21005-BR-1ng | SRR522075 | 0 | 0 | 0 |
| Human brain HCT20421-BR1ng | SRR522064 | 0 | 0 | 0 |
| Human brain HCT20413-BR1ng | SRR522063 | 0 | 0 | 0 |
| Human brain s_1_HCT21001-BR-10ng | SRR522068 | 0 | 1 | 1 |
| Human brain s_2_HCT21002-BR-10ng | SRR522071 | 0 | 1 | 1 |

**Table S2-2. (B)**

| MRE#2: 20:31385035-31385057 | | | |
| --- | --- | --- | --- |
| Description | Sample ID | MRE-containing | MRE-free |
| Universal human reference RNA s_8_HCT21008-UHR-1ng | SRR522148 | 0 | 1 |
| Universal human reference RNA s_7_HCT21007-UHR-1ng | SRR522145 | 2 | 0 |
| cell2_hESC | SRR522082 | 20 | 3 |
| cell1_hESC | SRR522081 | 3 | 0 |
| Human brain s_6_HCT21006-BR-1ng | SRR522077 | 0 | 0 |
| Human brain s_5_HCT21005-BR-1ng | SRR522075 | 0 | 0 |
| Human brain HCT20421-BR1ng | SRR522064 | 0 | 0 |
| Human brain HCT20413-BR1ng | SRR522063 | 0 | 1 |
| Human brain s_1_HCT21001-BR-10ng | SRR522068 | 0 | 0 |
| Human brain s_2_HCT21002-BR-10ng | SRR522071 | 0 | 1 |

Reference
