# Supplementary material for: Fine-tuning of microRNA-mediated repression of mRNA by splicing-regulated and highly repressive microRNA recognition element
Source: BMC Genomics. 2013 Jul 3;14:438. doi: 10.1186/1471-2164-14-438 (PMC3708814; doi:10.1186/1471-2164-14-438)
Supplement: Additional file 3 — Global trend of target proteins that were repressed or activated after transfection of hsa-miR-1 or hsa-miR-181a to HeLa cells. [file 1471-2164-14-438-S3.doc]

**Additional file 3**

**Global trend of target proteins that were repressed or activated after transfection of hsa-miR-1 or hsa-miR-181a to HeLa cells**

While using the proteomics data Baek *et al.* published in 2008, we have now shown that global analysis of multiple target proteins of a single miRNA (miR-124) supports the association of alternative splicing with highly repressive MREs. To show this association is not specific for miR-124, we have further analyzed the effects of miR-1 (**Table S3-1 and Figure S3-1**) and miR-181a (**Table S3-2 and Figure S3-2**). Selbach *et al.* also analyzed the effects of five individual miRNAs (let-7b, miR-1, miR-16, miR-30a and miR-155) on proteome using a similar approach. The study from Selbach *et al.* does not provide the seed information for each identified gene. As a result, similar analysis to the 5 microRNAs in Selbach *et al.*is not possible.

The fractions of highly repressive proteins (i.e., +30% repression) after transfection of hsa-miR-1 (**Table S3-1(B)**) or hsa-miR-181a (**Table S3-2(B)**) are smaller than hsa-miR-124 to HeLa cells, respectively (Supplementary Table S2(B)). The fractions of “Proteomics∩Seed(3'UTR)∩miRSVR∩AS”or “Proteomics∩Seed(ALL)∩miRSVR∩AS” are all significantly different from the“Proteomics∩Seed(X)”fraction in hsa-miR-1 (**Table S3-1(C)**) or hsa-miR-181a (**Table S3-2(C)**) (P<0.001, KS test). The “Proteomics∩Seed(ORF)∩miRSVR∩AS”are not significantly different from the“Proteomics∩Seed(X)”fraction in hsa-miR-1 (P=0.063, KS test) (**Table S3-1(C)**) but are significantly different from the“Proteomics∩Seed(X)”fraction in hsa-miR-181a (P=0.039, KS test) (**Table S3-2(C)**).

In summary, these results support the association between alternative splicing and highly repressive MRE.

**Supplementary table and figure legends**

**hsa-miR-1**

**Table S3-1.** (A) Definition and number of detected targets of various fractions after transfection of hsa-miR-1 to HeLa cells. (B) Numbers of genes with decreased or increased level caused by the expression of hsa-miR-1 in HeLa cells relative to mock control from various fractions. (C) Paired statistical analysis of various fractions. A P-value of under 0.05 is considered significant and indicated in red.

**Fig. S3-1.** hsa-miR-1 transfection significantly increases amounts of gene with AS event and seed-supported MREs. Cumulative curves present the cumulated fraction of target proteins that were repressed or activated after transfection of hsa-miR-124 to HeLa cells. The x-axis represents the protein output as a percentage of expression ratio change of the control HeLa cells. The y-axis represents the cumulative percentage. The text on the upper left corner presents the meaning of each curve. Proteomics means all the proteins that are differentially expressed upon the treatment with miR-124. Seed-free target genes are represented by seed(X). Target genes that have seeds found in the 3’UTR is represented by Seed(3’UTR). Target genes that have seeds found in the ORF is represented by Seed(ORF). Target genes that have seeds found in the full length is represented by Seed(ALL). The seeds detected by the miRSVR program is represented by miRSVR. The target proteins that are regulated by alternative splicing is represented by AS. All the data except for the AS information are taken from Baek, et al. (2008). (A) Comparison of all proteomics output with miRSVR with AS-supported proteomics output; (B) comparison of seed-free proteomics output with seed-in-ORF, miRSVR and AS-supported proteomics output; (C) comparison of seed-free proteomics output with seed-in 3’UTR, miRSVR and AS-supported proteomics output; (D) Comparison of seed-free proteomics output with seed-in-3’UTR or seed-in-ORF or seed-supported proteomics output, miRSVR and AS-supported proteomics output.

**hsa-miR-181a**

**Table S3-2.** (A) Definition and number of detected targets of various fractions after transfection of hsa-miR-181a to HeLa cells. (B) Numbers of genes with decreased or increased level caused by the expression of hsa-miR-1 in HeLa cells relative to mock control from various fractions. (C) Paired statistical analysis of various fractions. A P-value of under 0.05 is considered significant and indicated in red.

**Fig. S3-2.** hsa-miR-181a transfection significantly increases amounts of gene with AS event and seed-supported MREs. Cumulative curves present the cumulated fraction of target proteins that were repressed or activated after transfection of hsa-miR-124 to HeLa cells. The x-axis represents the protein output as a percentage of expression ratio change of the control HeLa cells. The y-axis represents the cumulative percentage. The text on the upper left corner presents the meaning of each curve. Proteomics means all the proteins that are differentially expressed upon the treatment with miR-124. Seed-free target genes are represented by seed(X). Target genes that have seeds found in the 3’UTR is represented by Seed(3’UTR). Target genes that have seeds found in the ORF is represented by Seed(ORF). Target genes that have seeds found in the full length is represented by Seed(ALL). The seeds detected by the miRSVR program is represented by miRSVR. The target proteins that are regulated by alternative splicing is represented by AS. All the data except for the AS information are taken from Baek, et al. (2008). (A) Comparison of all proteomics output with miRSVR with AS-supported proteomics output; (B) comparison of seed-free proteomics output with seed-in-ORF, miRSVR and AS-supported proteomics output; (C) comparison of seed-free proteomics output with seed-in 3’UTR, miRSVR and AS-supported proteomics output; (D) Comparison of seed-free proteomics output with seed-in-3’UTR or seed-in-ORF or seed-supported proteomics output, miRSVR and AS-supported proteomics output.

**hsa-miR-1**

**Table S3-1. (A)**


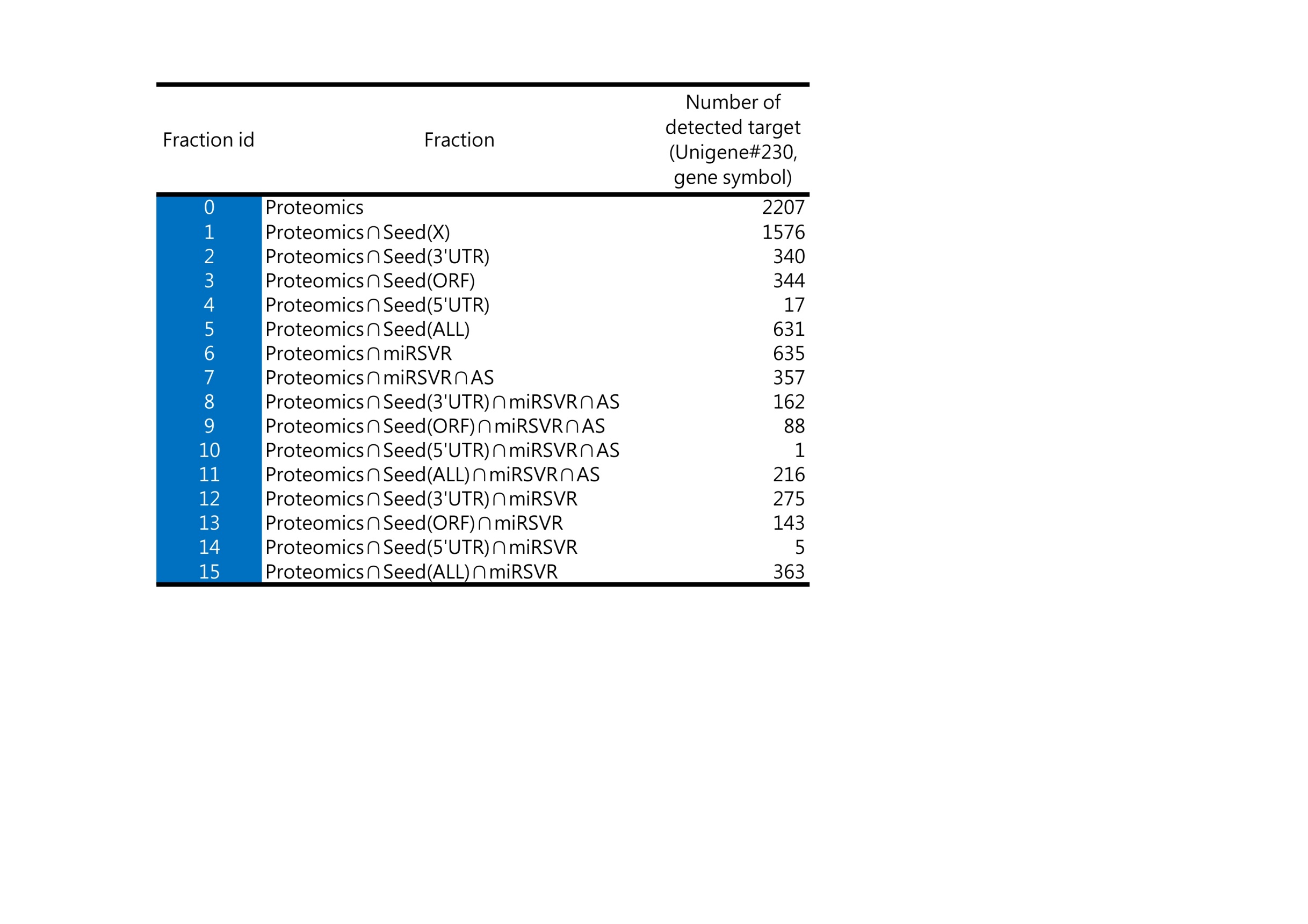


**Table S3-1. (B)**
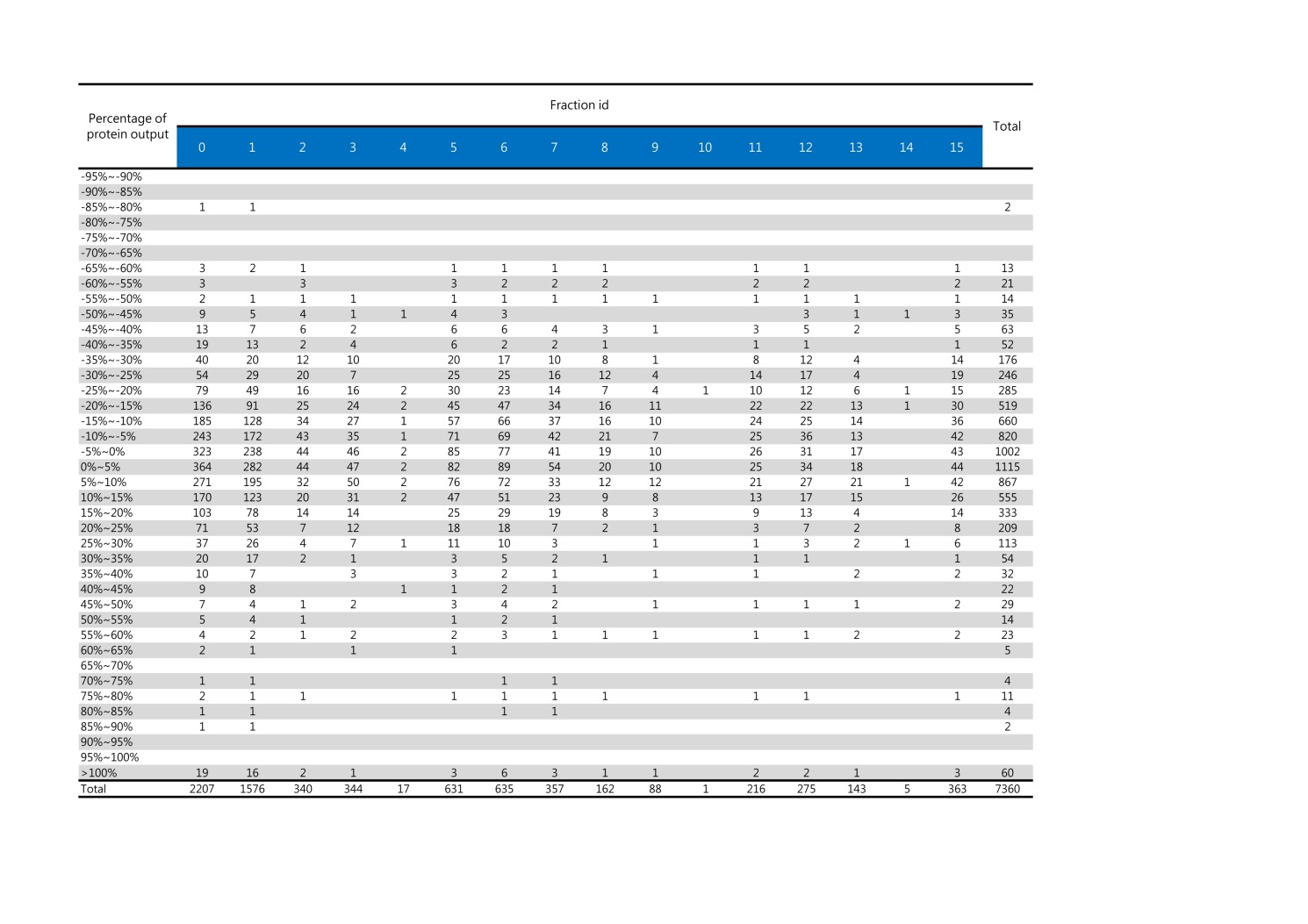


**Table S3-1. (C)**
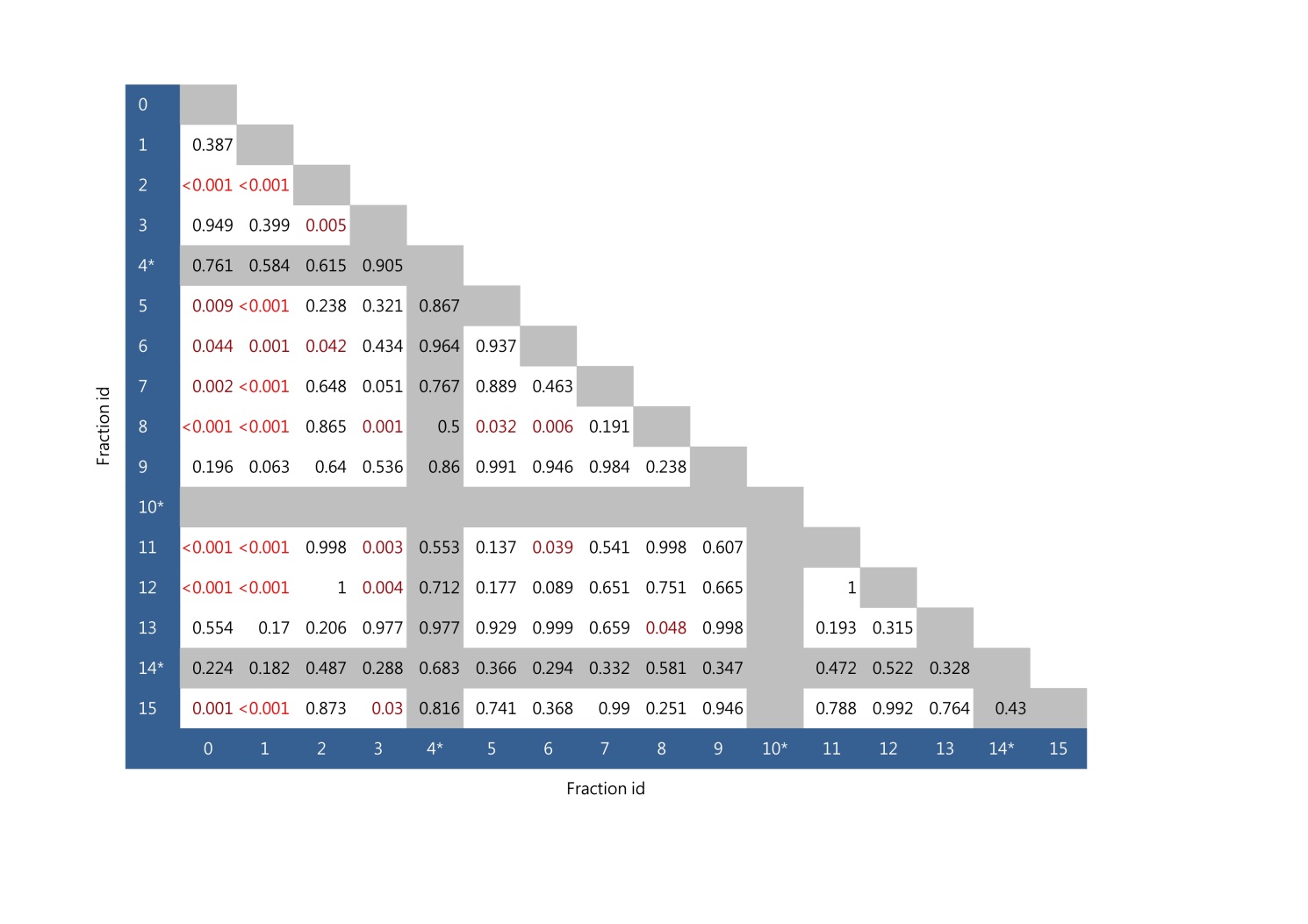


**Figure S3-1. (A)**
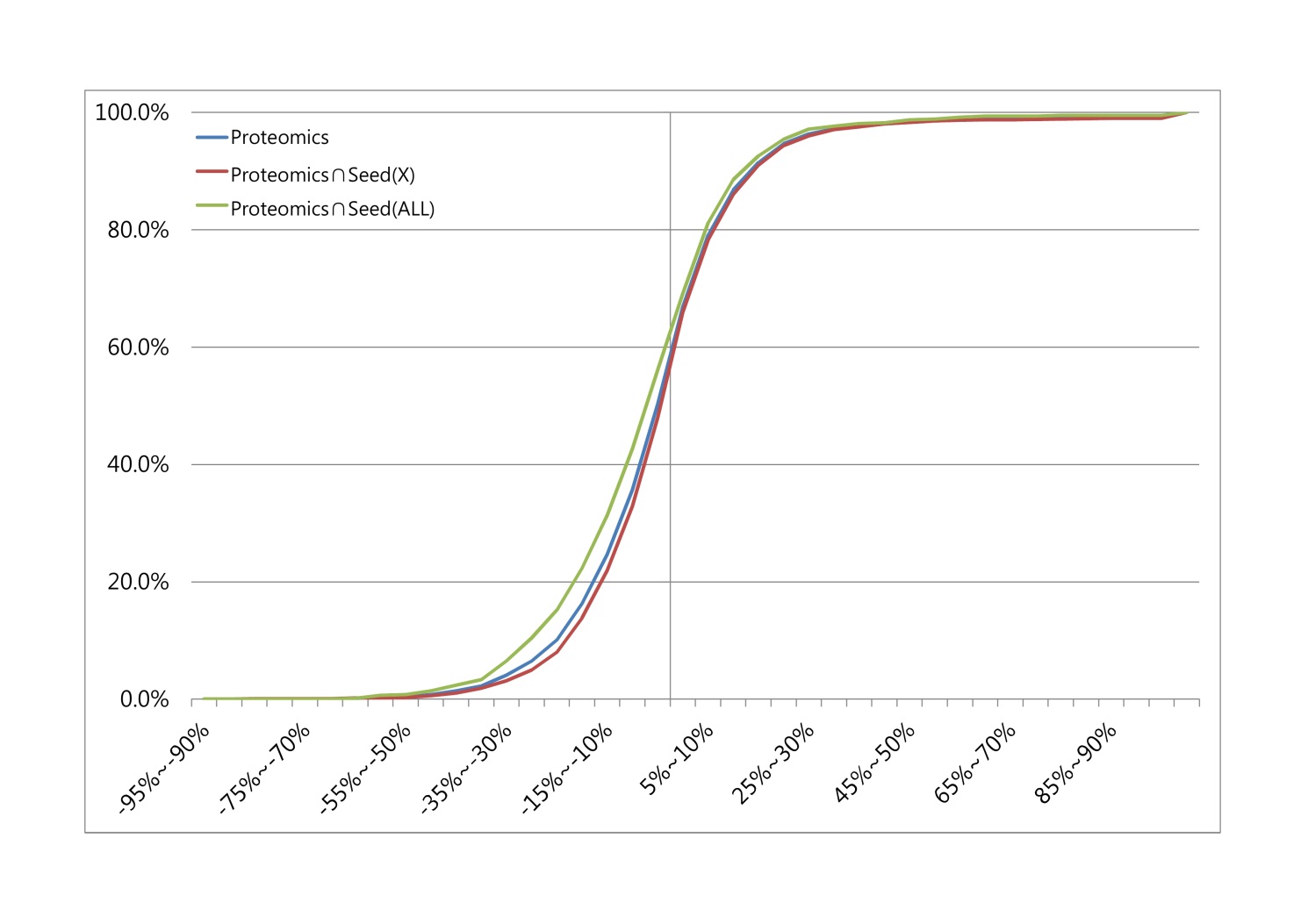


*Please refer to Table 1(C) to see the result of paired statistical analysis (K-S test).

**Figure S3-1. (B)**


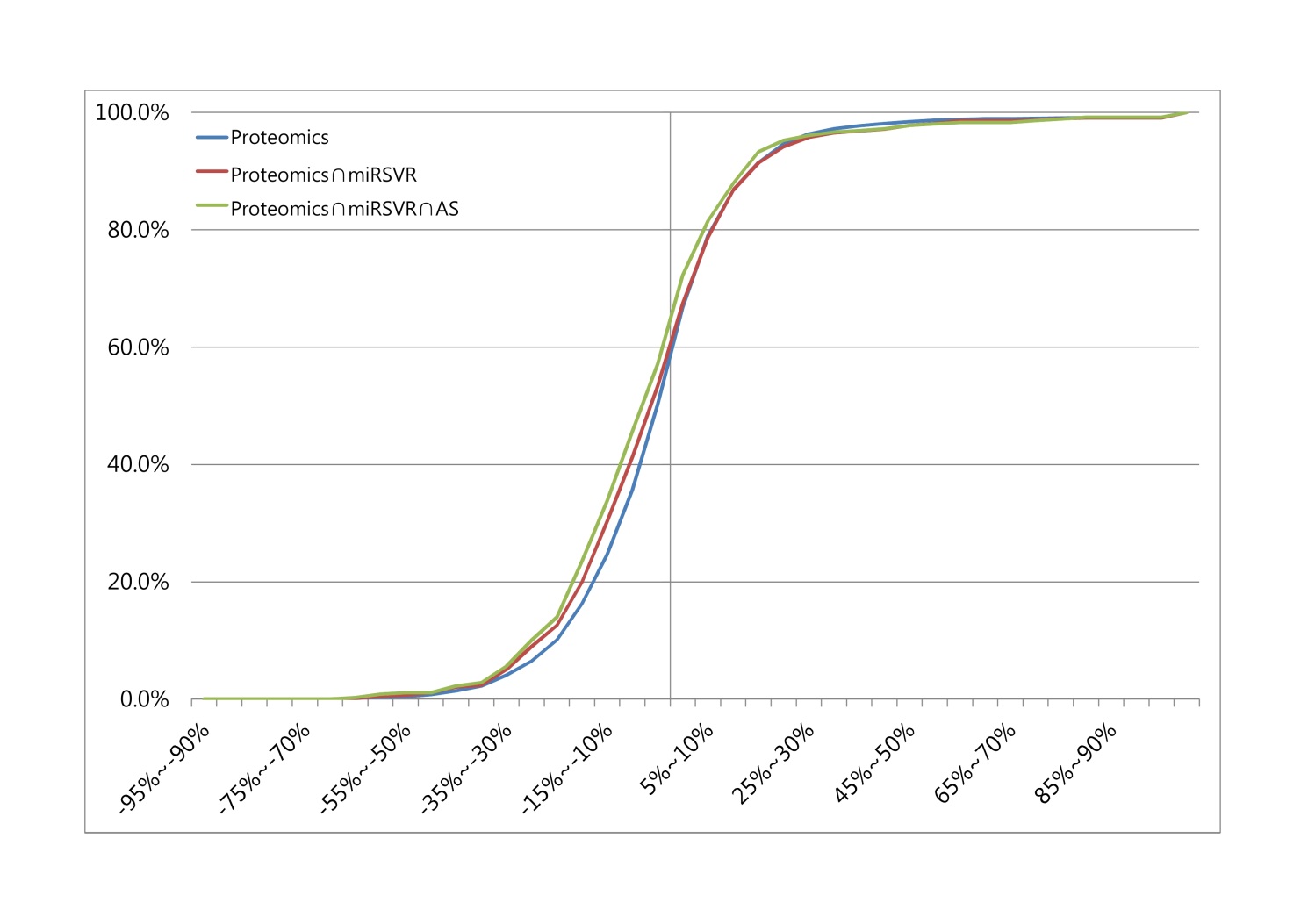


*Please refer to Table 1(C) to see the result of paired statistical analysis (K-S test).

**Figure S3-1. (C)**
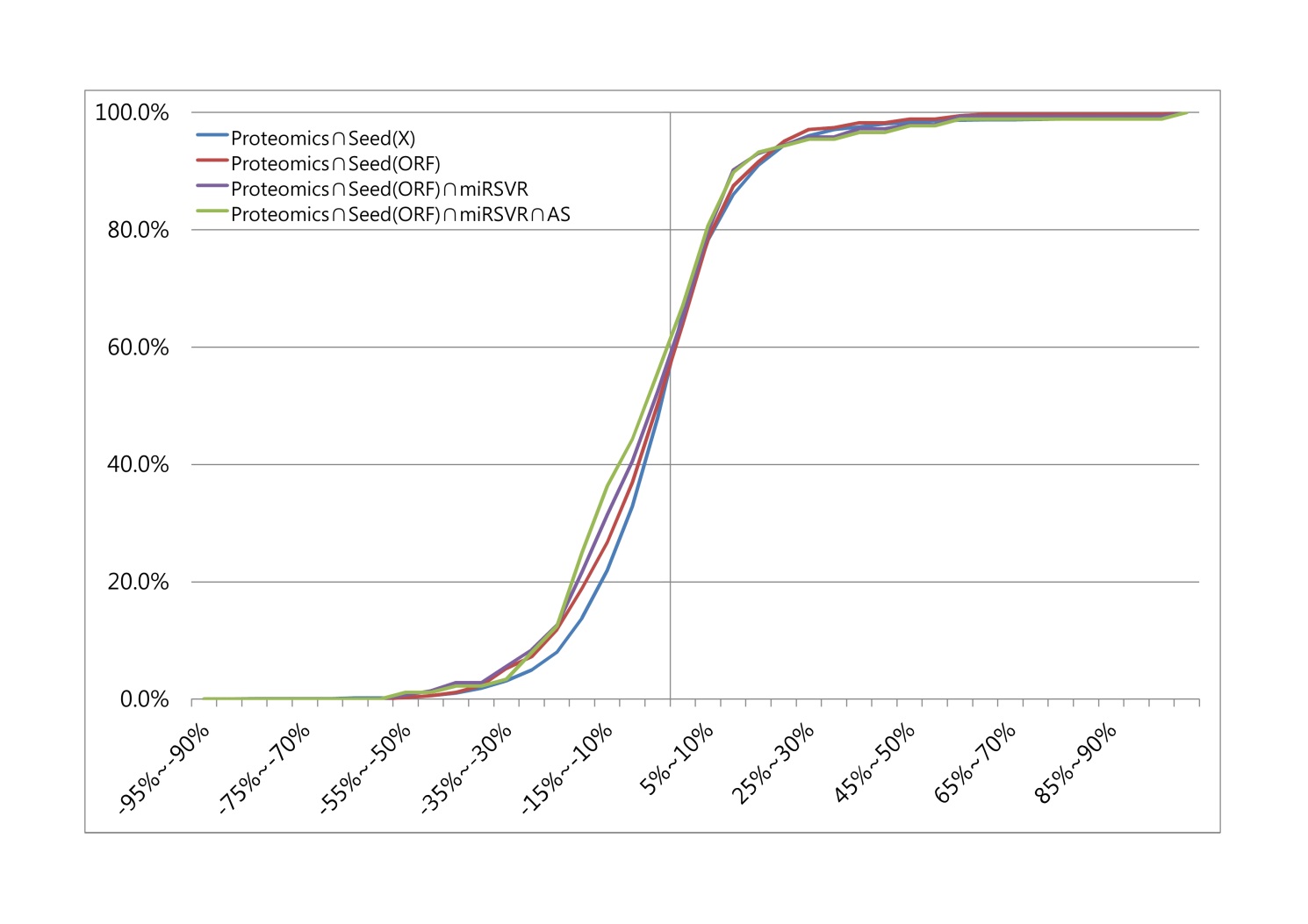


*Please refer to Table 1(C) to see the result of paired statistical analysis (K-S test).

**Figure S3-1. (D)**
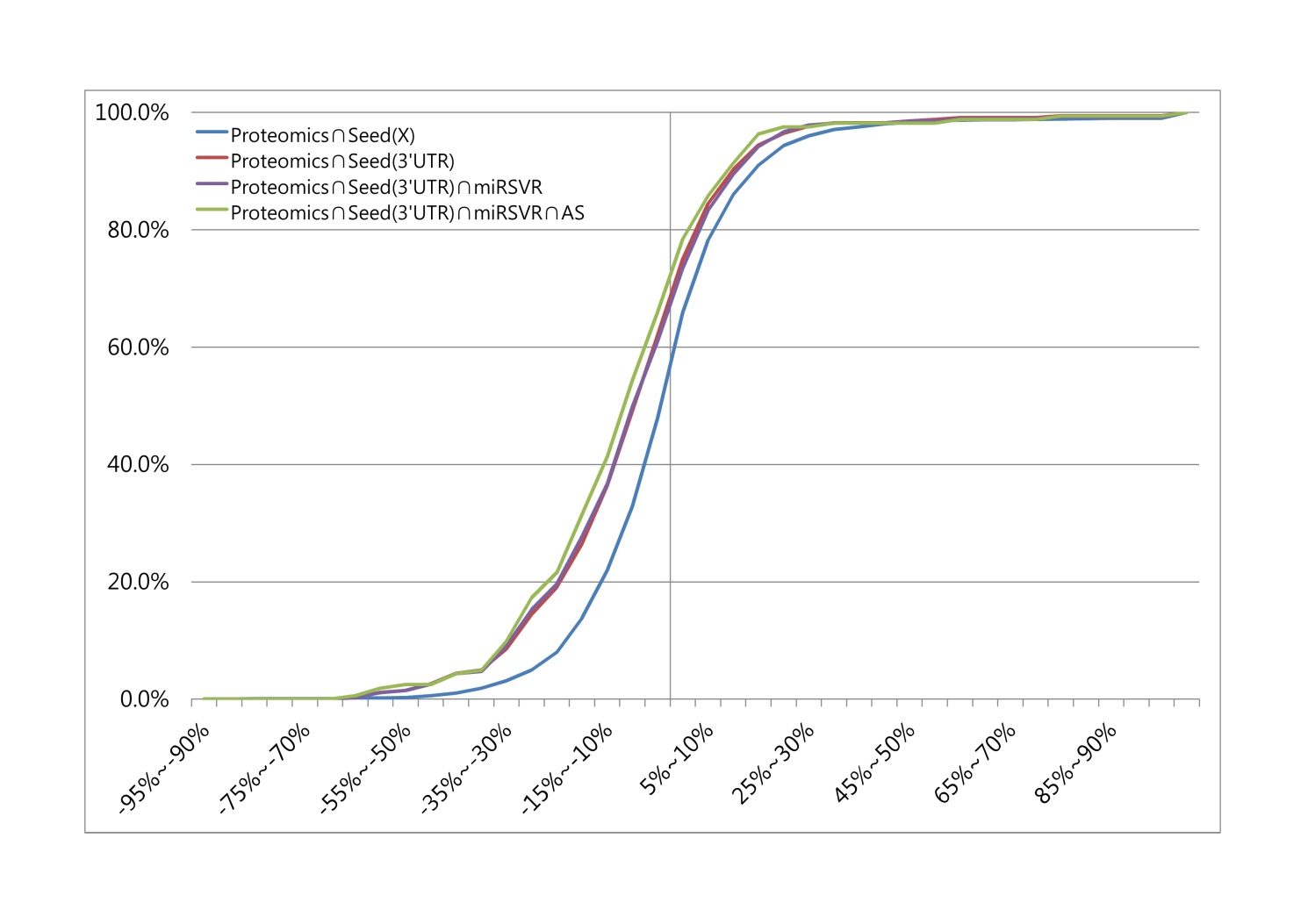


*Please refer to Table 1(C) to see the result of paired statistical analysis (K-S test).

**Figure S3-1. (E)**
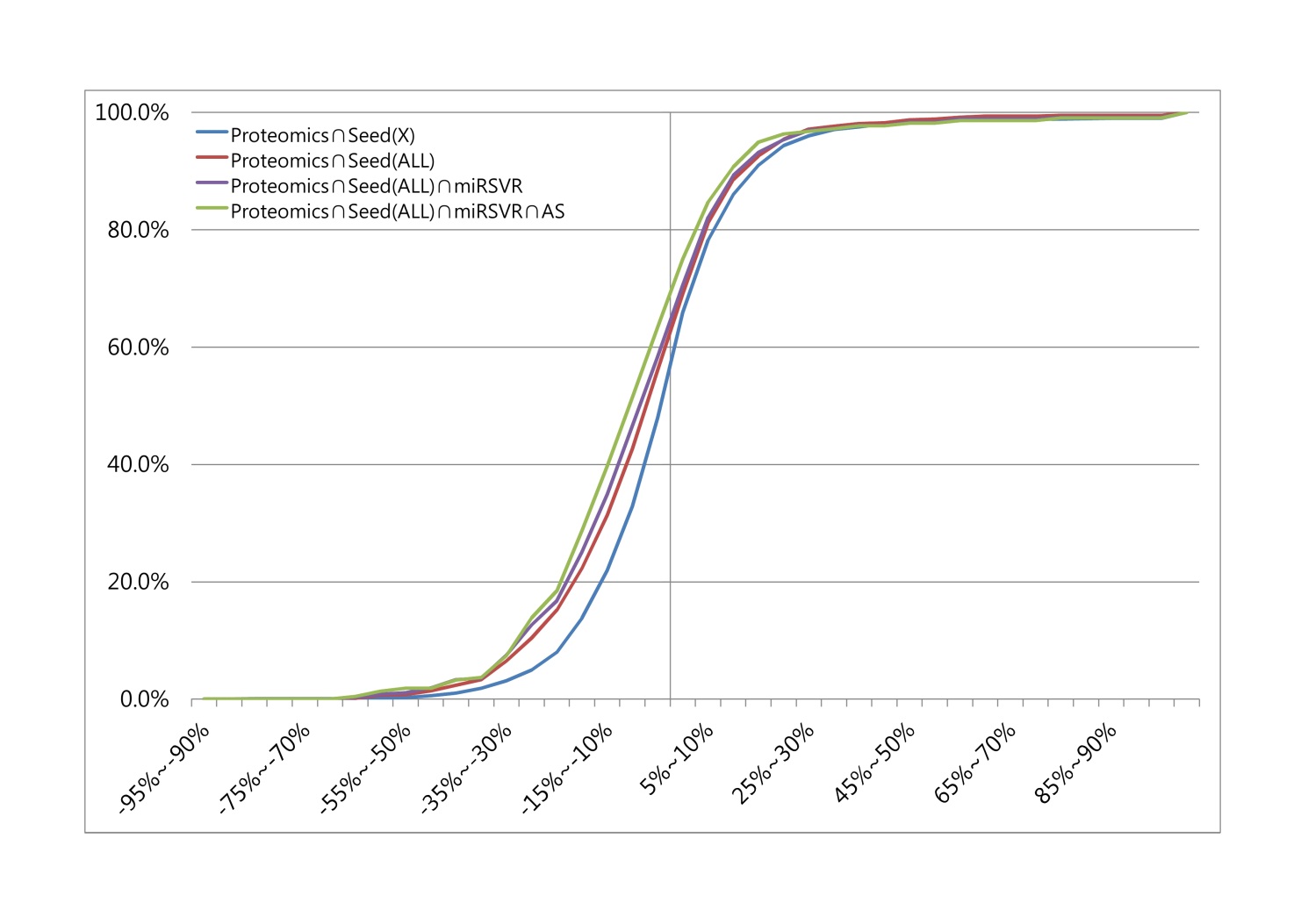


*Please refer to Table 1(C) to see the result of paired statistical analysis (K-S test).

**Figure S3-1. (F)**


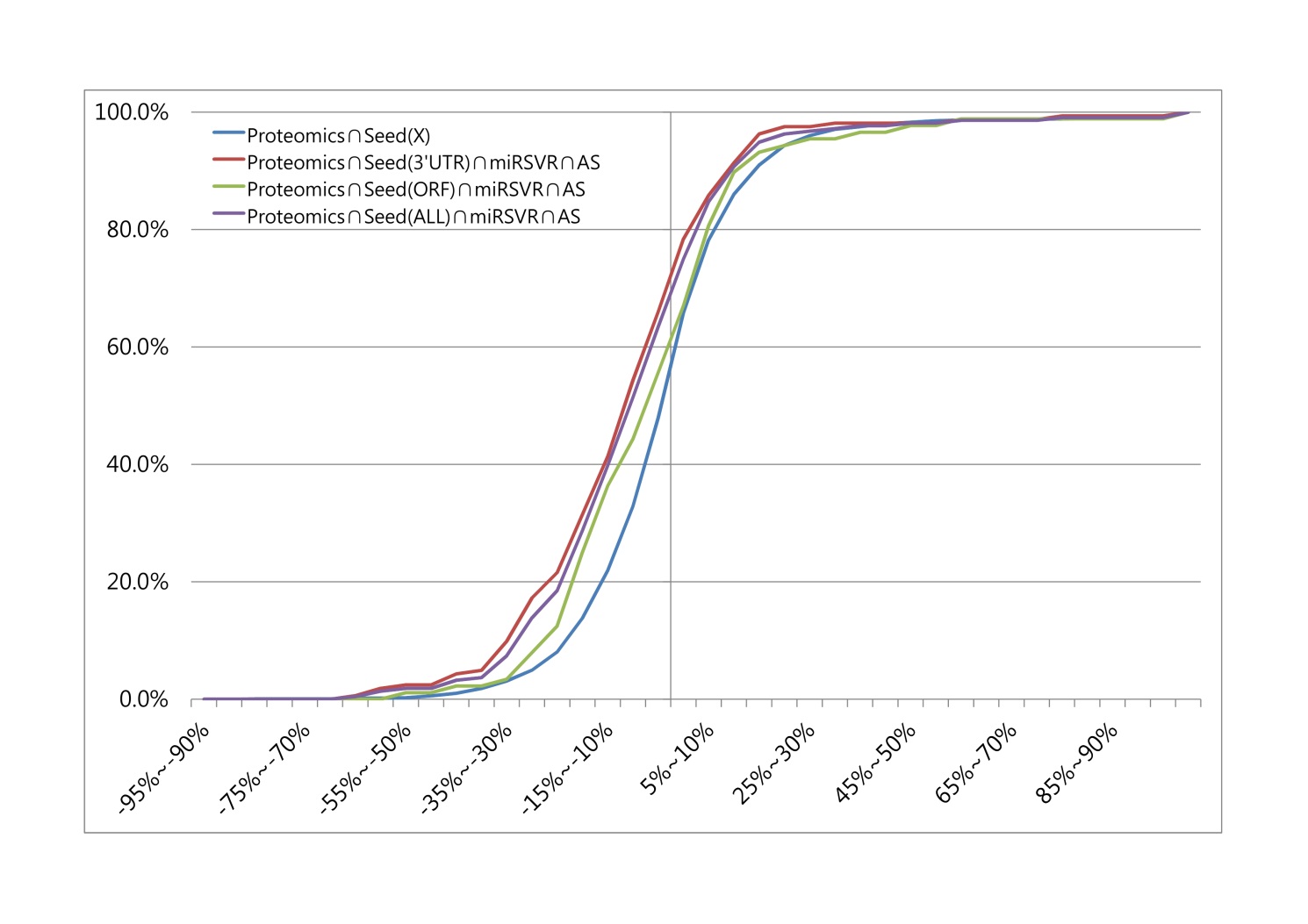


*Please refer to Table 1(C) to see the result of paired statistical analysis (K-S test).

**hsa-miR-181a**

**Table S3-2. (A)**


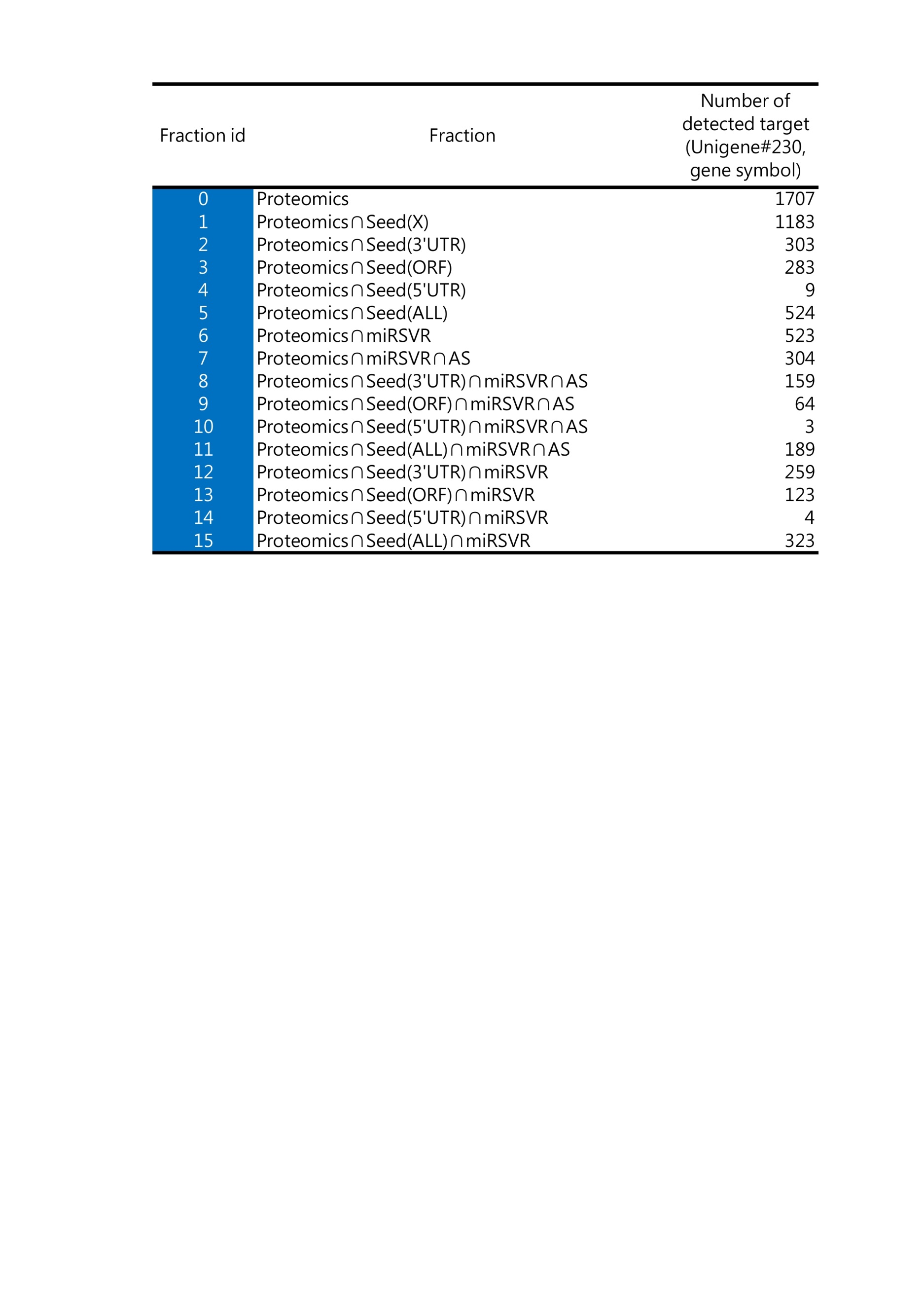


**Table S3-2. (B)**
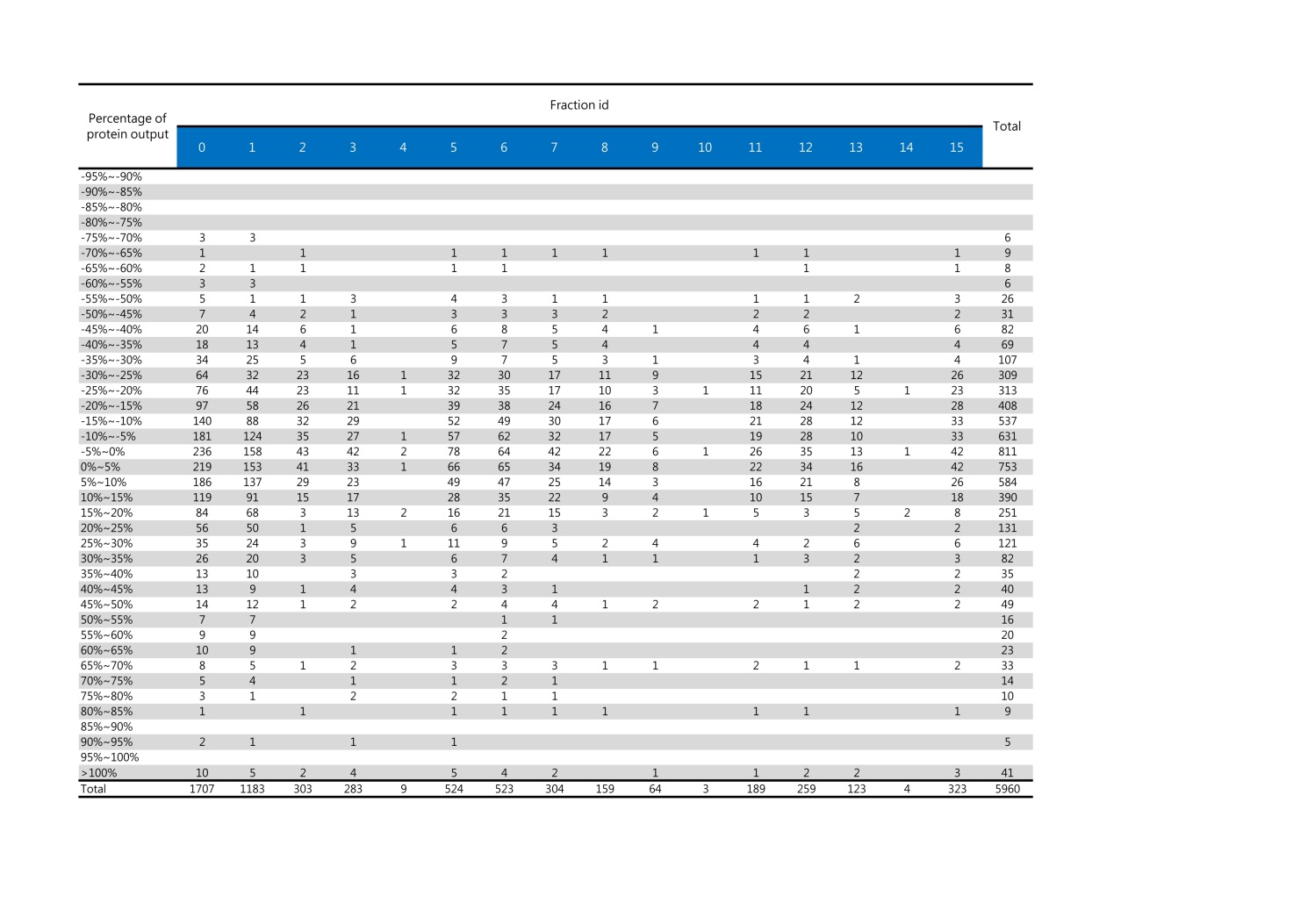


**Table S3-2. (C)**
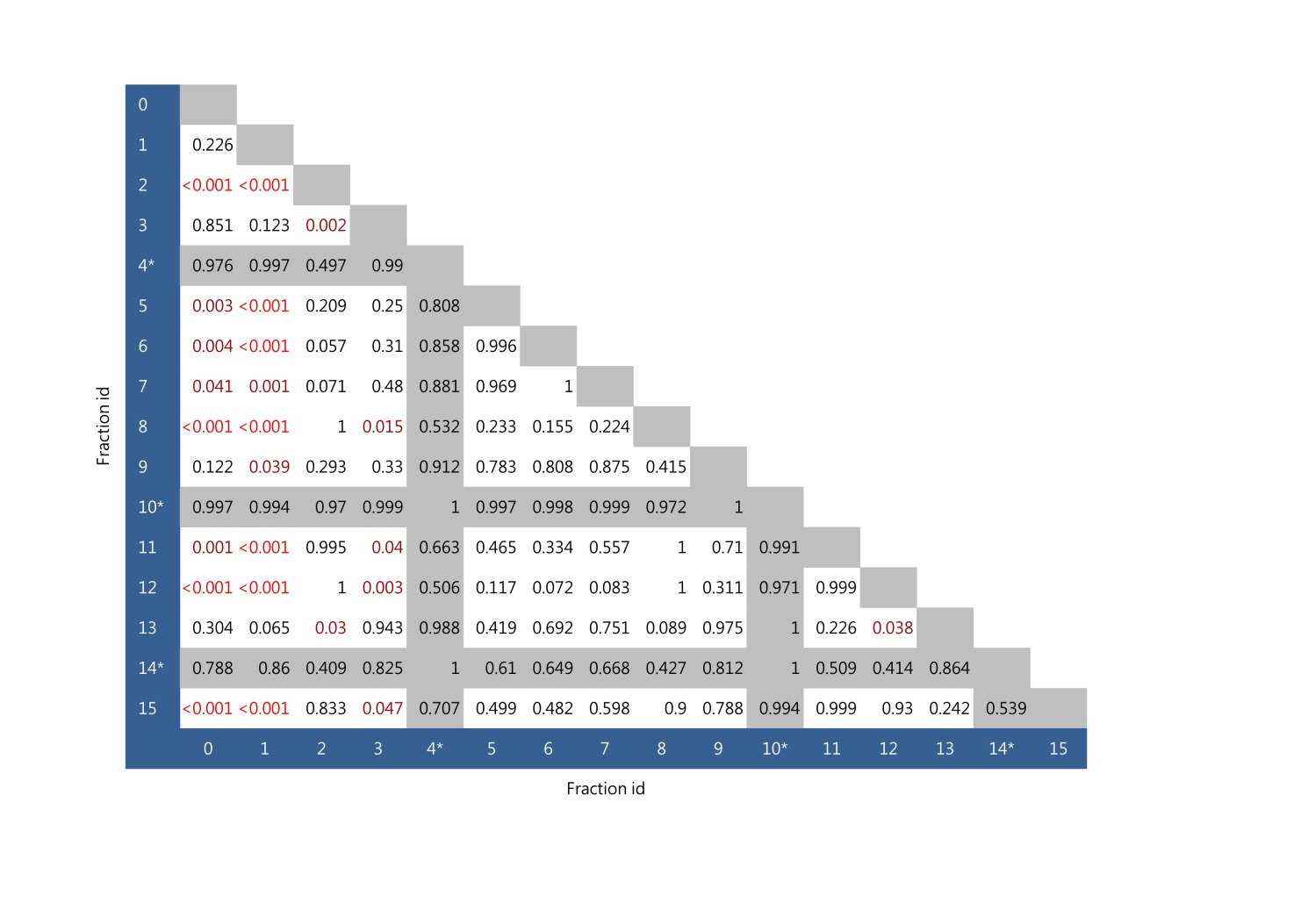


**Figure S3-2. (A)**
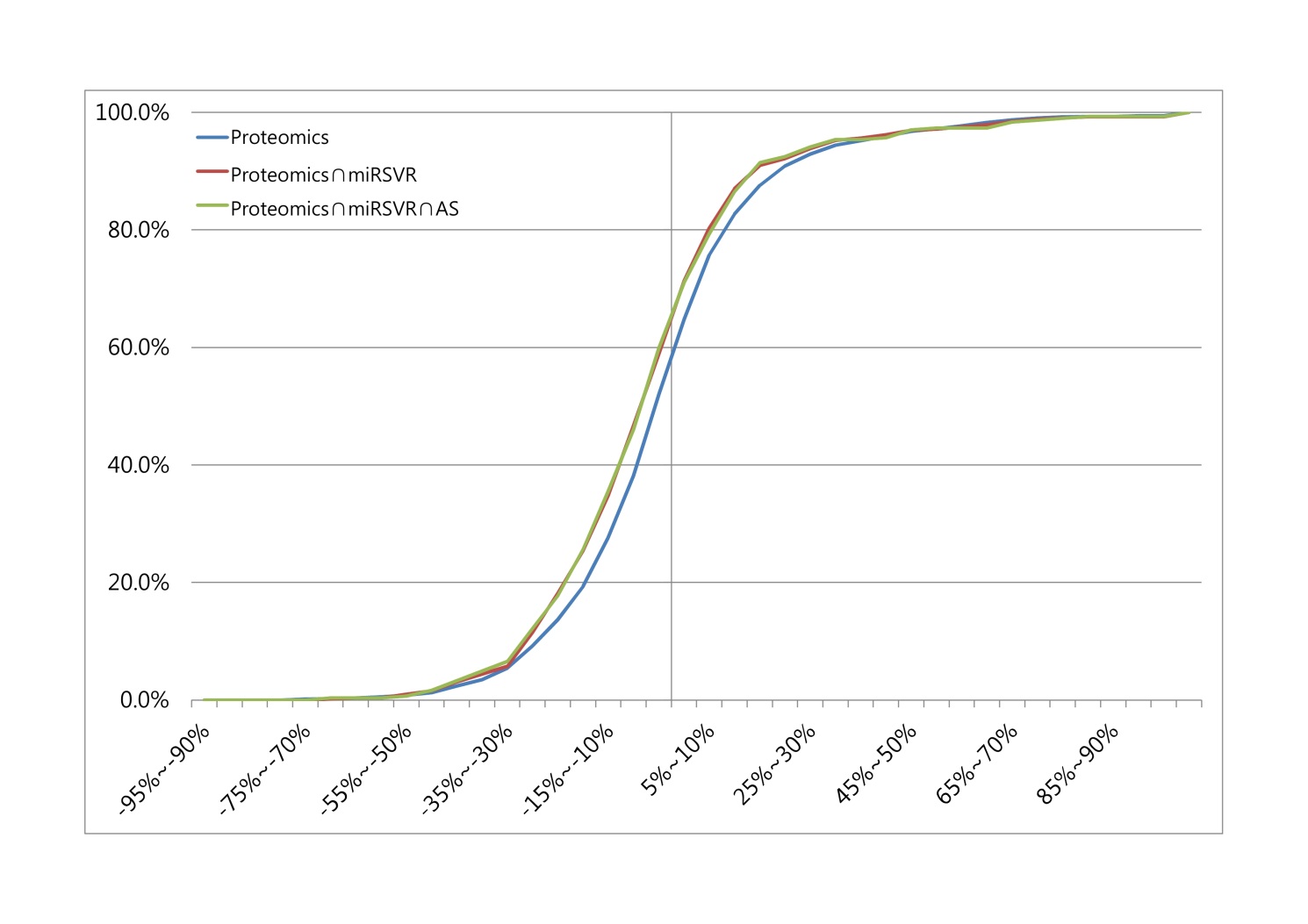


*Please refer to Table 2(C) to see the result of paired statistical analysis (K-S test).

**Figure S3-2. (B)**
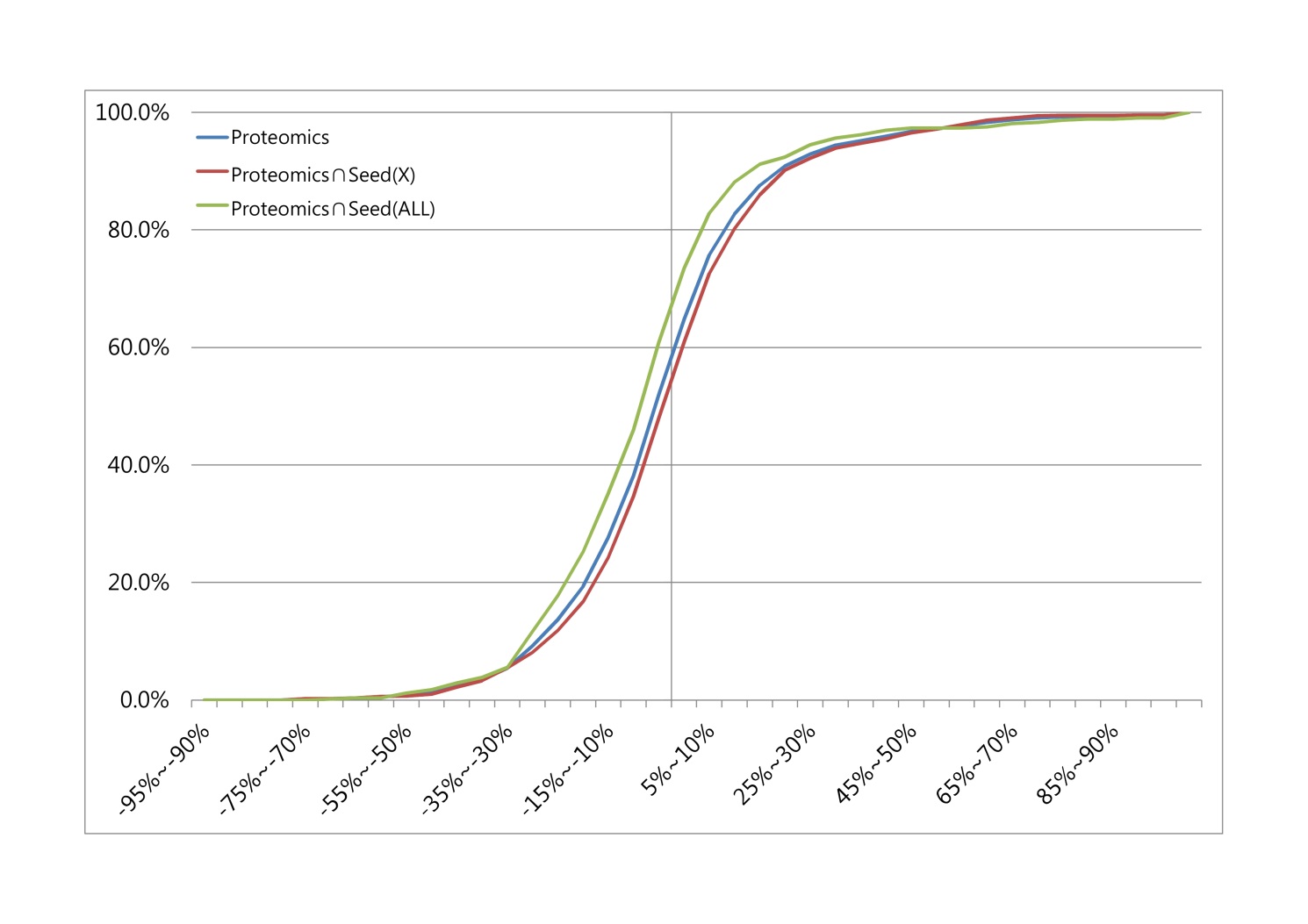


*Please refer to Table 2(C) to see the result of paired statistical analysis (K-S test).

**Figure S3-2. (C)**
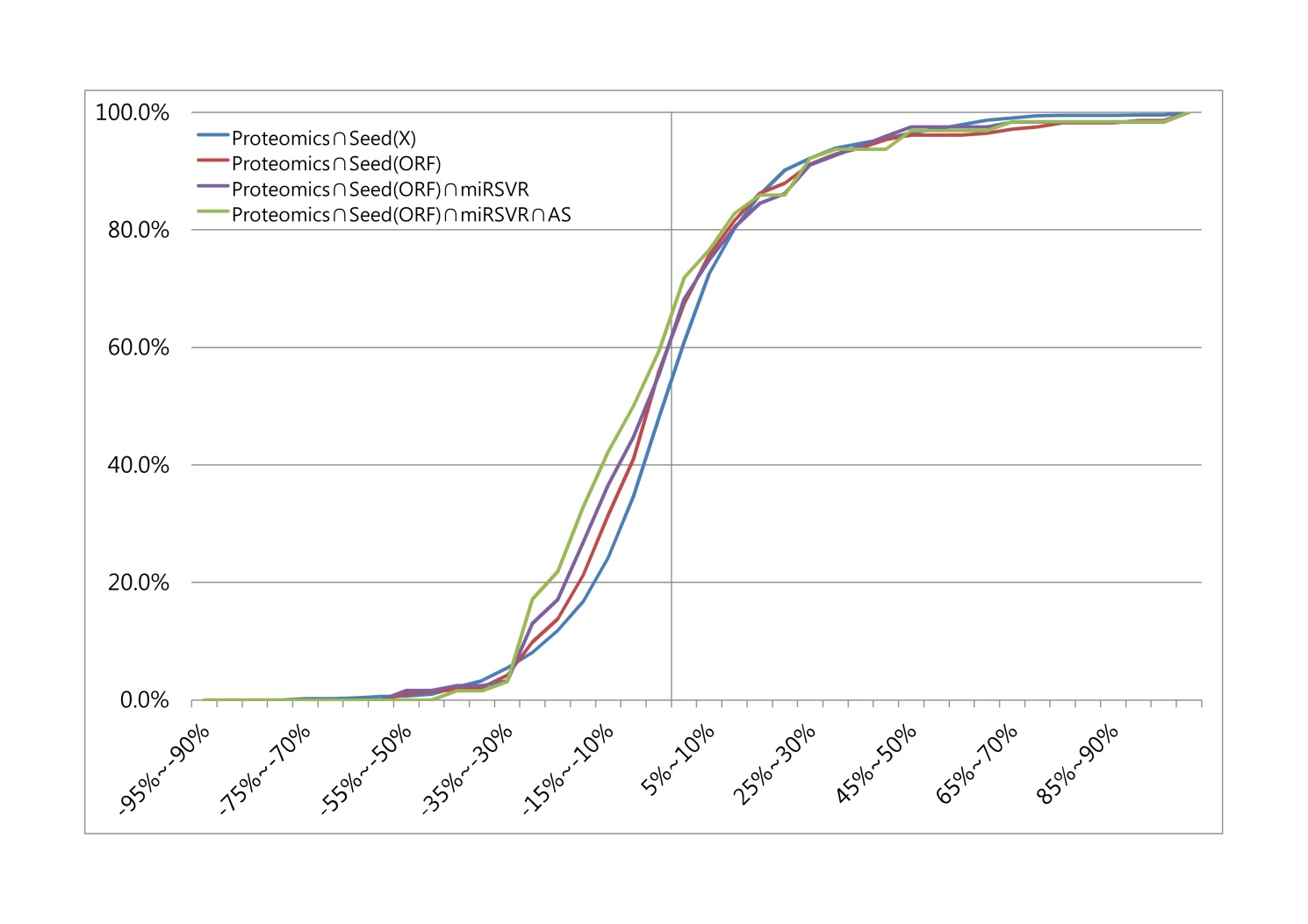


*Please refer to Table 2(C) to see the result of paired statistical analysis (K-S test).

**Figure S3-2. (D)**
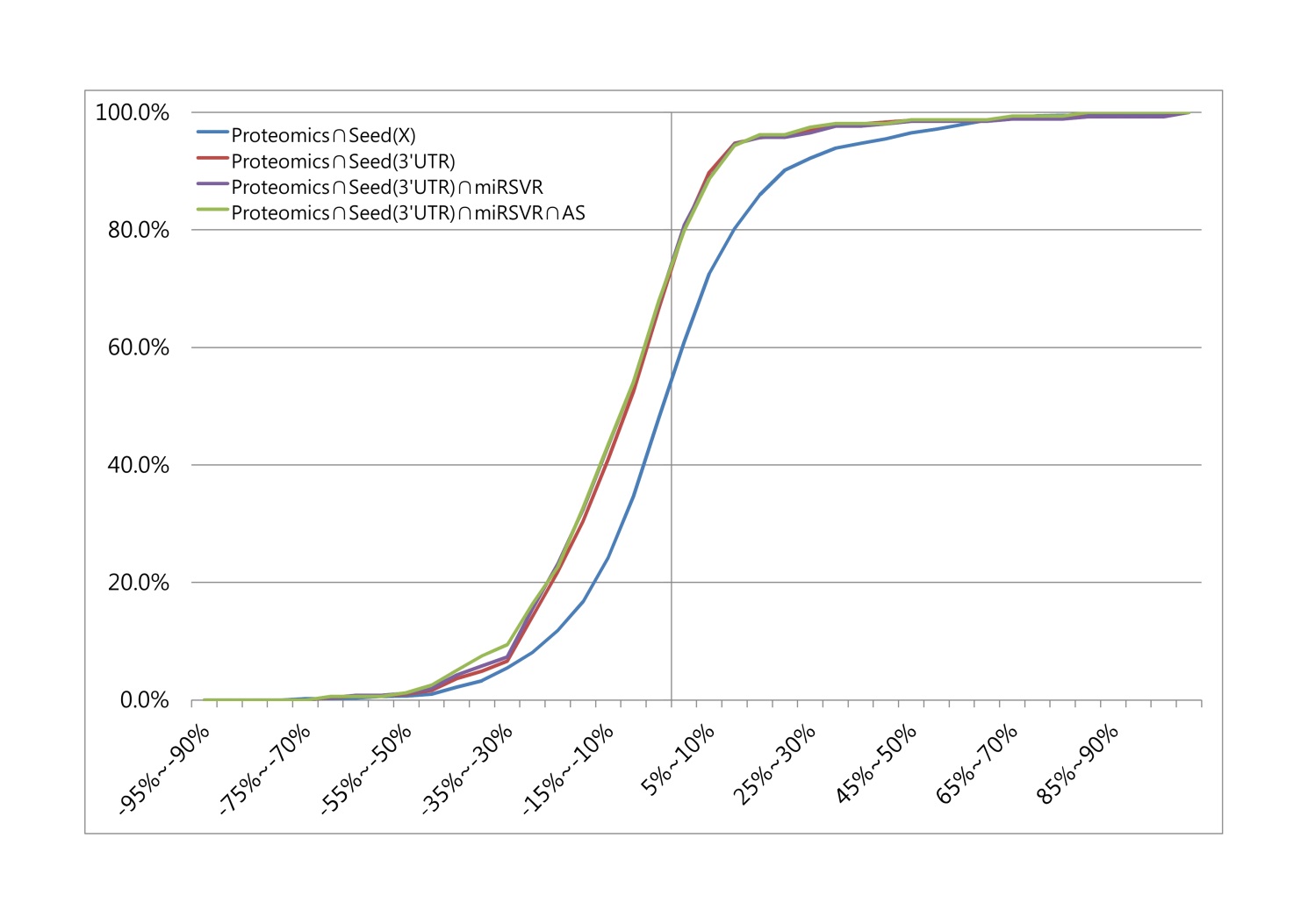


*Please refer to Table 2(C) to see the result of paired statistical analysis (K-S test).

**Figure S3-2. (E)**
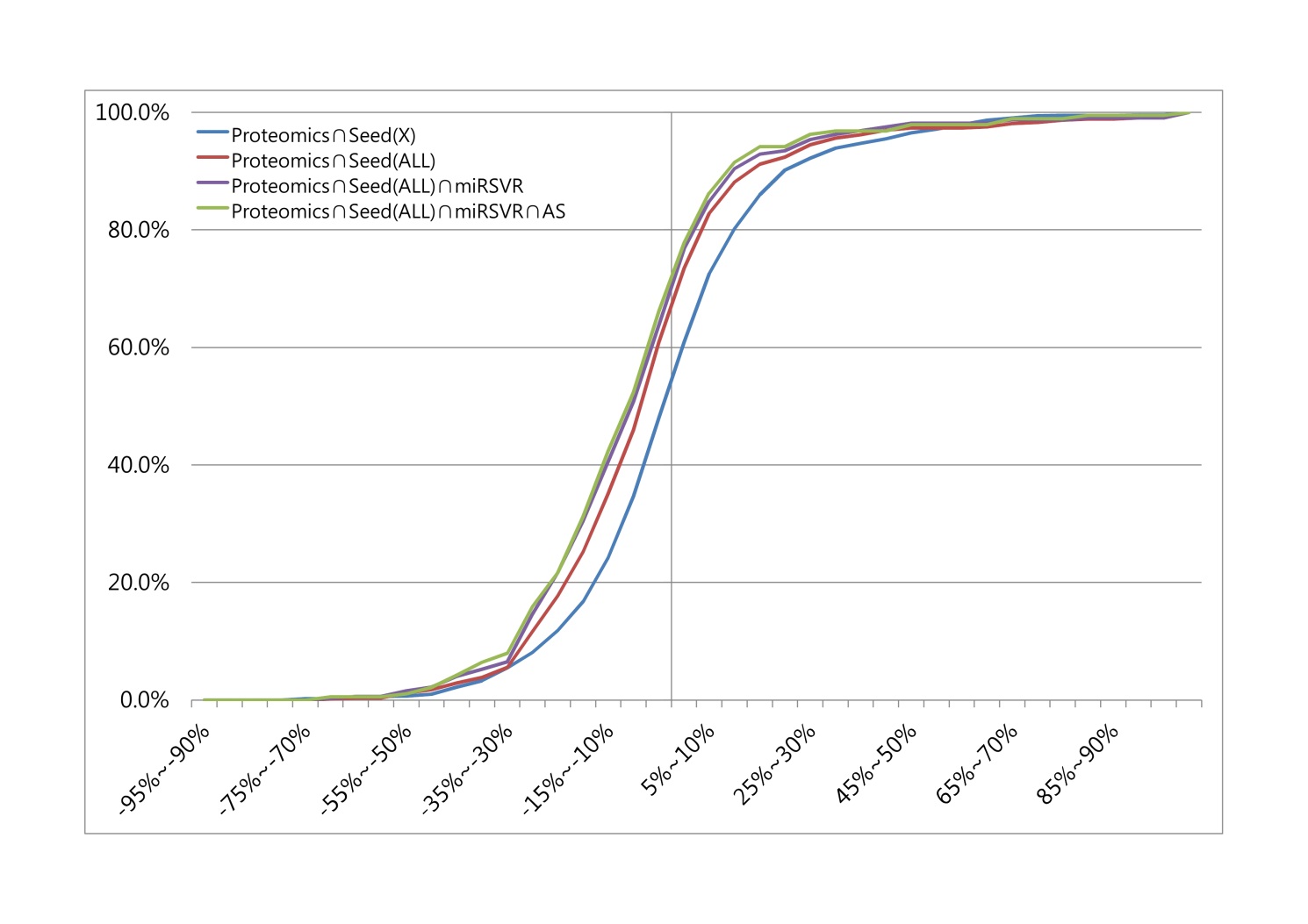


*Please refer to Table 2(C) to see the result of paired statistical analysis (K-S test).

**Figure S3-2. (F)**


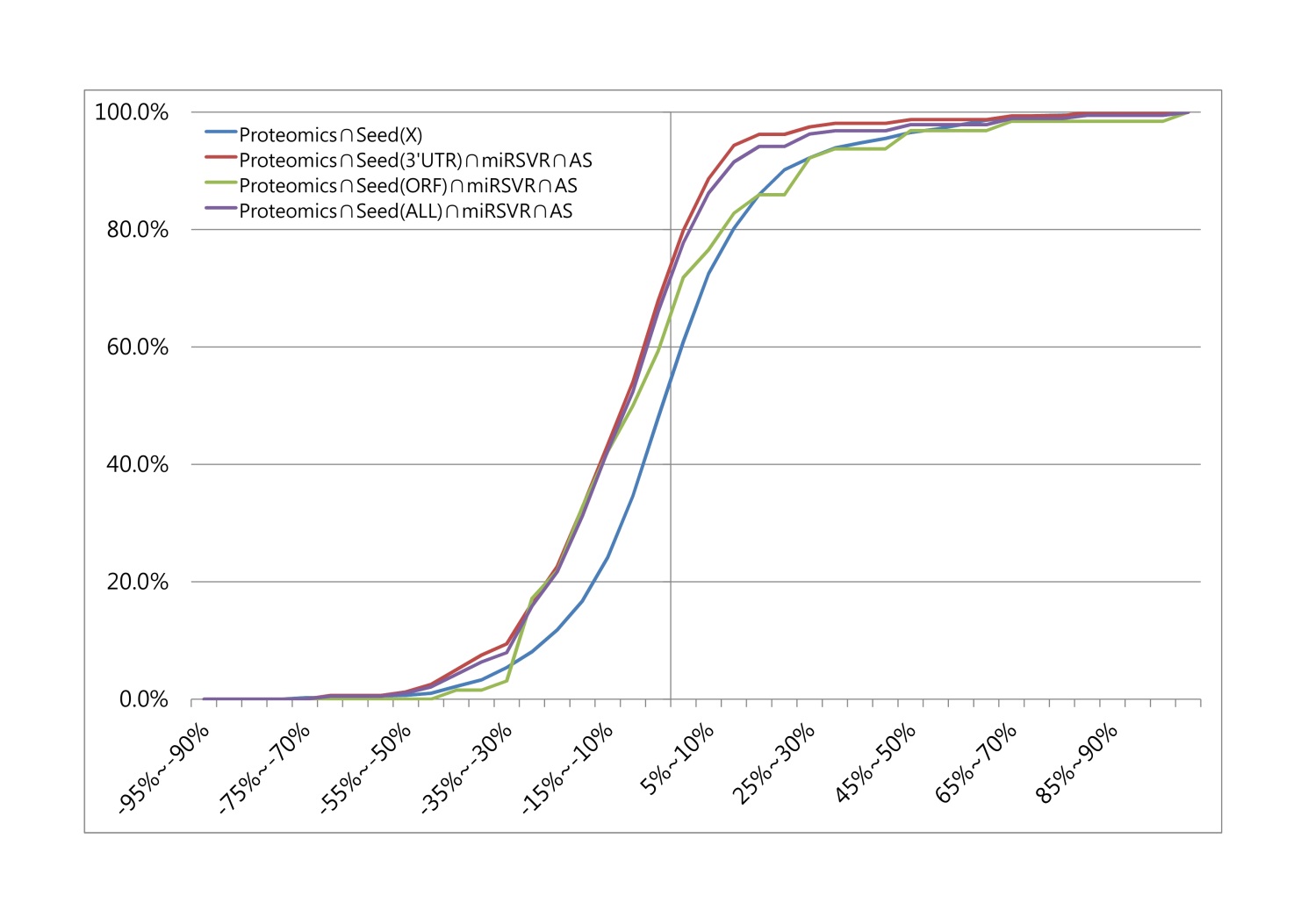


*Please refer to Table 2(C) to see the result of paired statistical analysis (K-S test).

Reference

1. Baek D, Villen J, Shin C, Camargo FD, Gygi SP, Bartel DP: **The impact of microRNAs on protein output**. *Nature* 2008, **455**(7209):64-71.

2. Selbach M, Schwanhausser B, Thierfelder N, Fang Z, Khanin R, Rajewsky N: **Widespread changes in protein synthesis induced by microRNAs**. *Nature* 2008, **455**(7209):58-63.
